# Oral or Topical Exposure to Glyphosate in Herbicide Formulation Impacts the Gut Microbiota and Survival Rates of Honey Bees

**DOI:** 10.1128/AEM.01150-20

**Published:** 2020-09-01

**Authors:** Erick V. S. Motta, Myra Mak, Tyler K. De Jong, J. Elijah Powell, Angela O'Donnell, Kristin J. Suhr, Ian M. Riddington, Nancy A. Moran

**Affiliations:** a Department of Integrative Biology, University of Texas at Austin, Austin, Texas, USA; b Mass Spectrometry Facility, Department of Chemistry, University of Texas at Austin, Austin, Texas, USA; University of Illinois at Chicago

**Keywords:** *Apis mellifera*, gut microbiome, herbicide, glyphosate

## Abstract

Honey bees are important agricultural pollinators that rely on a specific gut microbiota for the regulation of their immune system and defense against pathogens. Environmental stressors that affect the bee gut microbial community, such as antibiotics and glyphosate, can indirectly compromise bee health. Most of the experiments demonstrating these effects have been done under laboratory conditions with pure chemicals. Here, we investigated the oral and topical effects of various concentrations of glyphosate in a herbicide formulation on the honey bee gut microbiota and health under laboratory and field conditions. Under all of these conditions, the formulation, dissolved in sucrose syrup or water, affected the abundance of beneficial bacteria in the bee gut in a dose-dependent way. Mark-recapture experiments also demonstrated that bees exposed to the formulation were more likely to disappear from the colony, once reintroduced after exposure. Although no visible effects were observed for hives exposed to the formulation in field experiments, challenge trials with the pathogen *Serratia marcescens*, performed under laboratory conditions, revealed that bees from hives exposed to the formulation exhibited increased mortality compared with bees from control hives. In the field experiments, glyphosate was detected in honey collected from exposed hives, showing that worker bees transfer xenobiotics to the hive, thereby extending exposure and increasing the chances of exposure to recently emerged bees. These findings show that different routes of exposure to glyphosate-based herbicide can affect honey bees and their gut microbiota.

**IMPORTANCE** The honey bee gut microbial community plays a vital role in immune response and defense against opportunistic pathogens. Environmental stressors, such as the herbicide glyphosate, may affect the gut microbiota, with negative consequences for bee health. Glyphosate is usually sprayed in the field mixed with adjuvants, which enhance herbicidal activity. These adjuvants may also enhance undesired effects in nontargeted organisms. This seems to be the case for glyphosate-based herbicide on honey bees. As we show in this study, oral exposure to either pure glyphosate or glyphosate in a commercial herbicide formulation perturbs the gut microbiota of honey bees, and topical exposure to the formulation also has a direct effect on honey bee health, increasing mortality in a dose-dependent way and leaving surviving bees with a perturbed microbiota. Understanding the effects of herbicide formulations on honey bees may help to protect these important agricultural pollinators.

## Abstract (precis)

The honey bee gut microbial community plays a vital role in immune response and defense against opportunistic pathogens. Environmental stressors, such as the herbicide glyphosate, may affect the gut microbiota, with negative consequences for bee health. Glyphosate is usually sprayed in the field mixed with adjuvants, which enhance herbicidal activity. These adjuvants may also enhance undesired effects in nontargeted organisms. This seems to be the case for glyphosate-based herbicide on honey bees. As we show in this study, oral exposure to either pure glyphosate or glyphosate in a commercial herbicide formulation perturbs the gut microbiota of honey bees, and topical exposure to the formulation also has a direct effect on honey bee health, increasing mortality in a dose-dependent way and leaving surviving bees with a perturbed microbiota. Understanding the effects of herbicide formulations on honey bees may help to protect these important agricultural pollinators.

## INTRODUCTION

Honey bees are important agricultural pollinators whose populations have declined over the past decade. The reasons for colony failures are not fully understood but have been linked to environmental stressors, such as the spread of pathogens and parasites (1–6), reduction of food resources (7), and pesticide exposure (8–11). More recently, the herbicide glyphosate has been found to disrupt the gut microbiota of honey bees, reducing the abundance of beneficial bacterial species (12).

Glyphosate is the main active ingredient of many herbicide formulations used to kill unwanted vegetation not only in crop areas but also in nonagricultural settings, such as industrial sites, parks, railroads, roadsides, and recreational and residential areas (13). Its use is growing in connection with genetically engineered, herbicide-tolerant crops (14, 15). In such formulations, glyphosate is found in its salt form, which affects its absorption by the targeted organism. Different salt forms of glyphosate are applied in the field (16), along with surfactants, such as polyethylated tallow amine, to enhance herbicide efficacy (17). These glyphosate-based formulations are commercially available at different concentrations which reach up to 48% (wt/vol) of glyphosate as the main active ingredient, based on product labeling. These formulations are usually recommended to be diluted in water before spraying on target plants, with concentrations ranging from 0.4% to 7% glyphosate. Once the formulation is inside the plant, it is the glyphosate acid that binds to the target enzyme in susceptible plants and causes the herbicidal effect.

Glyphosate inhibits the 5-enolpyruvyl-shikimate-3-phosphate synthase (EPSPS) in the shikimate pathway. This stops the production of essential aromatic metabolites, such as aromatic amino acids (phenylalanine, tryptophan, and tyrosine), folate cofactors, benzoid and naphtoid coenzymes, phenazines, siderophores, and others (18). A deficit in aromatic amino acids leads to a reduction in protein synthesis and, ultimately, to the organism’s death. All plants and some microorganisms, but not animals, contain a functional shikimate pathway and therefore are potentially susceptible to glyphosate. Thus, glyphosate can only be used in genetically engineered crops carrying a tolerant version of EPSPS, which is commonly derived from *Agrobacterium* spp. (19).

EPSPS enzymes from different organisms are classified as class Iα, Iβ, or II based on their biochemical properties and phylogenetic distinctions. Class Iα enzymes are naturally sensitive to low concentrations of glyphosate and occur naturally in all plants and some *Bacteria*, whereas class Iβ enzymes are found in *Archaea* (20). On the other hand, class II enzymes usually tolerate higher doses of glyphosate than class I enzymes and in nature occur exclusively in *Bacteria* (21). Moreover, classes I and II EPSPS diverge by more than 30% in amino acid sequences (22). Previous studies have shown that most bee gut bacterial species carry a functional shikimate pathway with a class Iα or a class II EPSPS, whereas others lack this enzyme, suggesting that some bee gut bacteria are selectively inhibited by glyphosate (12).

Honey bees have coevolved with a beneficial, specialized, and socially transmitted gut microbiota, comprised of five to eight dominant bacterial members (23). These members belong to different taxa, including *Snodgrassella* (24), *Gilliamella* (24), *Bifidobacterium* (25), *Lactobacillus* Firm-4 (26), *Lactobacillus* Firm-5 (26–28), *Bartonella* (29), *Frischella* (30), and *Commensalibacter*. These bacterial taxa are specialized and diverse in terms of metabolic capabilities, i.e., they inhabit specific niches and play specific roles in the bee gut (23, 31, 32). For example, *Snodgrassella alvi* forms a biofilm layer in the ileum (33) and stimulates the host immune system (34). *Snodgrassella alvi* is also involved in cross-feeding interactions with other bacteria, such as *Gilliamella* spp. (28, 35), which in turn detoxifies the gut environment by metabolizing toxic sugars (36) and helps in digestion of recalcitrant components of the bee diet along with *Bifidobacterium* spp. (35). *Lactobacillus* spp. acidify the bee gut, potentially inhibiting the proliferation of some opportunistic pathogens (37). The microbiome as a whole promotes host weight gain (38) and regulates immune signaling pathways (34, 39). Regarding glyphosate susceptibility, some bee gut bacterial species contain a functional shikimate pathway and carry either a susceptible, class Iα EPSPS (*Snodgrassella*, *Gilliamella*, *Frischella*, and *Bifidobacterium*) or a tolerant, class II EPSPS (*Bartonella*), whereas other bacteria contain a truncated shikimate pathway with a class II EPSPS (*Lactobacillus* Firm-4) or lack the gene that encodes EPSPS (*Lactobacillus* Firm-5) (12). In the absence of a functional shikimate pathway, these *Lactobacillus* bacteria may rely on the uptake of aromatic amino acids from the gut environment, which may come from the bee diet or from other gut bacteria that produce these metabolites.

Honey bees can be directly exposed to high concentrations of glyphosate and other components of the formulation when foraging during herbicide application (up to 2.0 g/liter) or when collecting pollen (up 629 mg/kg) and nectar (up to 31.3 mg/kg) from plants that have been recently treated with the herbicide (40). They can also be exposed to residues of glyphosate when collecting contaminated water (up to 3.1 mg/liter) (41–43). Moreover, glyphosate residues have been detected in commercialized and natural honey (up to 0.3 mg/liter) (44–47) and even in larval bees (up to 19.50 mg/kg) (40).

Recent studies demonstrating that the honey bee gut microbiota is affected by exposure to glyphosate were mostly performed with the pure chemical and under laboratory conditions (12, 48, 49). Commercial formulations usually contain adjuvants, such as surfactants, to enhance herbicidal efficacy. Since honey bees can be exposed to glyphosate when collecting contaminated nectar, pollen, and water sources or when foraging during herbicide application, we decided to investigate whether the microbial perturbations observed with pure glyphosate would also be observed when bees are orally or topically exposed to glyphosate in herbicide formulations. Moreover, we investigated the impacts of the same formulation on the bee gut microbiota under field conditions, under a worst-case scenario in which bees are directly exposed to the formulation. Our findings suggest that oral or topical exposure to glyphosate, pure or in herbicide formulation, can affect the honey bee gut microbiota under laboratory and field conditions.

## RESULTS

### Oral exposure of honey bees to glyphosate, pure or in herbicide formulation, under laboratory conditions—effects on gut microbial composition.

**FIG 1:**
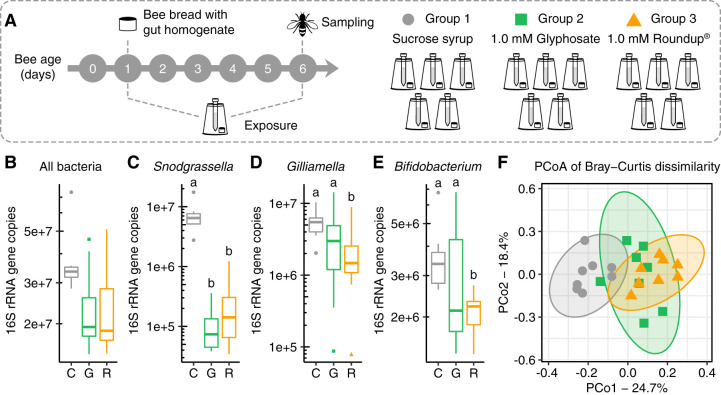
The effects of glyphosate and Roundup formulation on the honey bee gut microbiota. (A) Newly emerged workers were exposed to 1.0 mM glyphosate in sucrose syrup, 1.0 mM Roundup in sucrose syrup, or only sucrose syrup for 5 days. (B to E) Box plots of absolute abundance of total bacteria (B), *Snodgrassella alvi* (C), *Gilliamella* spp. (D), and *Bifidobacterium* spp. (E) in the guts of bees sampled from control, glyphosate, and Roundup groups (*n* = 8 for each group). Groups with distinct letters are statistically different (*P* < 0.05, Kruskal-Wallis test followed by Dunn’s multiple-comparison test). (F) Principal coordinate analysis of Bray-Curtis dissimilarity of gut community compositions of control, glyphosate, and Roundup groups.

Age-controlled bees raised under laboratory conditions were divided into three groups which were fed either sucrose syrup, 1.0 mM glyphosate dissolved in sucrose syrup, or Roundup formulation corresponding to 1.0 mM glyphosate dissolved in sucrose syrup (Fig. 1A). After 5 days of exposure, bees were sampled, and their microbial communities were evaluated by extracting DNA from their guts and performing 16S rRNA amplicon sequencing and qPCR analyses. From these data, we obtained estimates of absolute abundances for the main bacterial taxa in control and treatment bees.

Both glyphosate and Roundup formulation affected the abundance of beneficial bacteria in the guts of honey bees (Fig. 1B) by significantly decreasing the absolute abundance of *Snodgrassella alvi* compared with the control group (Fig. 1C, Fig. S1). The absolute abundances of *Gilliamella* spp. and *Bifidobacterium* spp. also decreased in exposed bees, but this decrease was only significant in the group exposed to the Roundup formulation (Fig. 1D and E).

Moreover, principal coordinate analysis of gut community compositions demonstrated that bees treated with pure glyphosate or Roundup formulation clustered together and apart from those of controls; this was true for analyses based on both Bray-Curtis dissimilarities, which reflect relative abundances (Fig. 1F; see Table S1 in the supplemental material), and weighted UniFrac dissimilarities, which include phylogenetic relatedness (50) (see Fig. S1 in the supplemental material; Table S1). These results support and extend previous work showing that glyphosate perturbs the bee gut microbiota by reducing the abundance of beneficial bacterial species (12); glyphosate-based formulations have similar effects.

### Oral exposure of honey bees to glyphosate in herbicide formulation—effects on hive recovery rates and gut microbial composition.

Considering the effects observed for both glyphosate and Roundup formulation on the honey bee gut microbiota, we decided to conduct more experiments with the formulation. Based on recommended applications, the concentrations used for weed control vary according to location and type of weed, ranging from 0.4% to 7.0% Roundup formulation. We tested a concentration of 0.1% Roundup formulation, which is lower than the minimum concentration recommended to spray in the field and is in the same magnitude of glyphosate concentrations detected in pollen and nectar from recently exposed plants (40).

**FIG 2:**
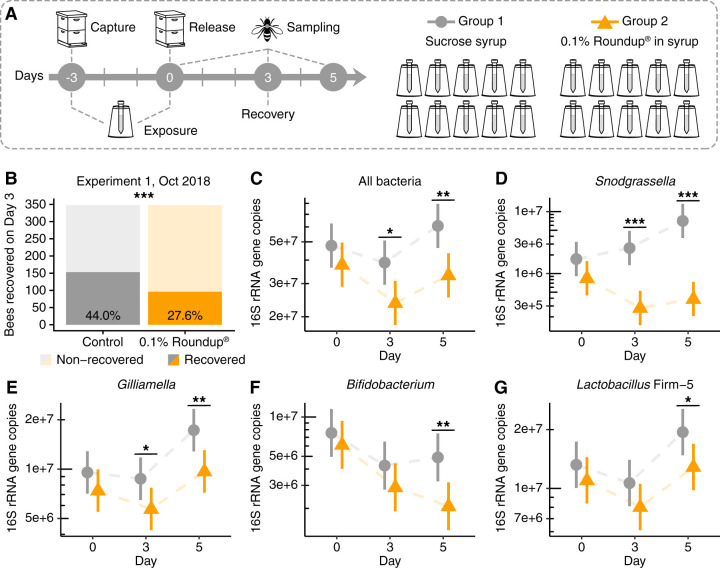
Recovery rates and gut microbial changes for honey bees returned to the hive after oral exposure to 0.1% Roundup formulation. (A) Experimental design. (B) Number of worker bees recovered from the hive at day 3 posttreatment (***, *P* < 0.001, chi-squared test). (C to G) Scatterplots of bacterial abundances in the guts of control and treatment bees sampled at days 0, 3, and 5 posttreatment (*n* = 15 for each group and time point), with error bars indicating 95% confidence intervals. Total 16S rRNA gene copies were estimated by qPCR and corrected for rRNA operon number per genome. Generalized linear mixed-effects models assuming Poisson regression were used to compare changes in bacterial abundances between control and treatment bees per sampling time. Mixed models were fitted using the package lme4 and followed by *post hoc* tests using package emmeans (86). *, *P* < 0.05; **, *P* < 0.01; and ***, *P* < 0.001.

In fall 2018, hundreds of worker bees were collected from inside a hive and divided into two groups, namely, control and treatment, which were fed sterile sucrose syrup or 0.1% Roundup dissolved in sucrose syrup, respectively (Fig. 2A). These bees were marked on the thorax with different paint colors, white or pink, respectively, and 3 days after treatment under laboratory conditions, the bees were reintroduced to their hive. Three days after hive reintroduction, all remaining marked bees were recovered; only 27.6% of Roundup-treated bees were recaptured, which was significantly lower than the 44.0% of control bees recaptured (Fig. 2B).

We also sampled control and treatment bees throughout this experiment at the end of treatment (day 0 posttreatment), at the time of recovery (day 3 posttreatment), and 2 days after the recovery time (day 5 posttreatment), and we evaluated their gut microbial compositions by 16S rRNA amplicon sequencing and qPCR analyses. Overall, treated bees exhibited a significant reduction in the absolute abundance of gut bacteria compared with control bees at days 3 and 5 posttreatment (Fig. 2C). This decrease in total bacterial abundance was accompanied by a significant reduction in the absolute abundance of the core bacterial members *Snodgrassella alvi* (Fig. 2D) and *Gilliamella* spp. (Fig. 2E) at days 3 and 5 posttreatment, as well as *Bifidobacterium* spp. (Fig. 2F) and *Lactobacillus* Firm-5 (Fig. 2G) at day 5 posttreatment. As found for the laboratory experiment described above, a principal coordinate analysis of community compositions based on 16S rRNA amplicons demonstrated that treated bees diverged from controls at days 3 and 5 posttreatment, and this result was supported by both Bray-Curtis or weighted UniFrac dissimilarities (50) (see Fig. S2 in the supplemental material; see Table S2 in the supplemental material).

This recovery experiment was replicated four more times using bees collected from different hives in spring or summer 2019 (see Fig. S3 in the supplemental material; see Table S3 in the supplemental material). Two of these experiments found significant decreases in recovery rates for treatment groups (Fig. S3A and G), but two others found no significant differences between control and treatment recovery rates (Fig. S3D and F). We also performed a color-bias validation experiment, which showed no significant difference in recovery between bees marked either pink or white, the colors used in these experiments (Fig. S3H).

We also processed bees from control and treatment groups from two more replicate experiments. This time, the abundances of total bacteria and/or *Snodgrassella alvi* were measured by qPCR (Fig. S3). In the second experiment, which showed significant differences in recovery rates between groups, we observed similar decreases in abundance for *Snodgrassella alvi* in treated bees at both days 0 and 3 posttreatment (Fig. S3C), but not for total bacteria (Fig. S3B). In the third experiment, in which we did not see a significant difference in recovery rates between control and treatment bees, we also did not observe significant changes in *Snodgrassella alvi* abundance after treatment (Fig. S3E).

### Oral exposure of honey bees to glyphosate in a herbicide formulation under field conditions.

**(i) Effects on the gut microbiota.** In parallel with the previously described experiments, we conducted more experiments to evaluate the potential effects of Roundup exposure to honey bees under field conditions. Honey bee hives were established at two sites on private land in Driftwood, TX, in 2018 and 2019, in both cases approximately 2 months before starting experiments. The first field experiment was performed at site 1 in August/September 2018, whereas the second field experiment was performed at sites 1 and 2 in August/September 2019.

**FIG 3:**
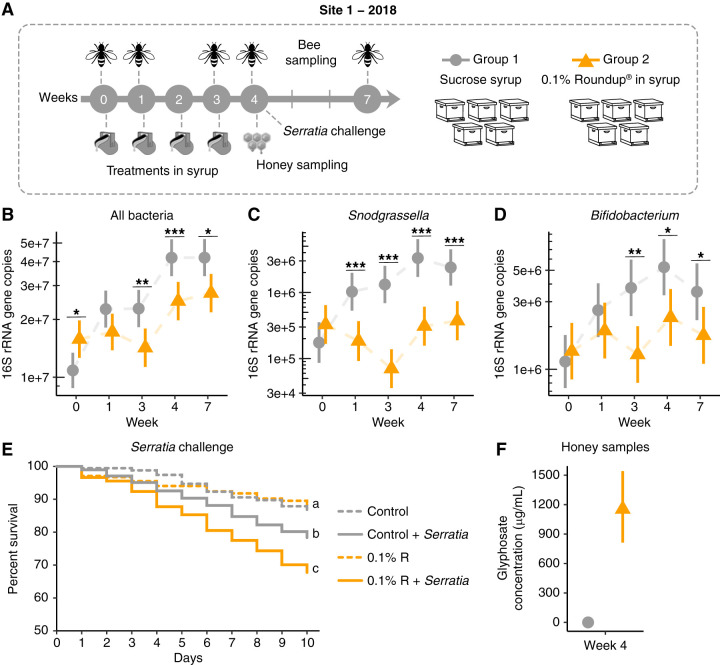
Gut microbial changes and susceptibility to bacterial infections in honey bees from hives exposed to Roundup formulation in site 1 in 2018. (A) Field experiment performed in site 1 in 2018. Ten hives were split into 2 groups to be exposed to 0.5 liters of sucrose syrup or 0.1% Roundup formulation dissolved in sucrose syrup at weeks 0, 1, 2, and 3. Treatment was placed in a reservoir inside the hives to avoid cross-contamination. Bees were sampled at weeks 0, 1, 3, 4, and 7. Uncapped honey samples were collected at week 4. (B to D) Scatterplots showing abundances of total bacteria (B), *Snodgrassella alvi* (C), and *Bifidobacterium* spp. (D) in the guts of bees sampled from control (sucrose syrup) and treatment (0.1% Roundup in syrup) groups on weeks 0, 1, 3, 4, and 7, with error bars indicating 95% confidence intervals (*n* = 5 hives per group, 15 bees per hive) per sampling time. Generalized linear mixed-effects models assuming Poisson regression were used to compare changes in bacterial abundances between control and treatment groups. Mixed models were fitted using the package lme4 (85) and followed by *post hoc* tests using the package emmeans (86). *, *P* < 0.05; **, *P* < 0.01; and ***, *P* < 0.001. (E) Survival rates of worker bees after Serratia marcescens kz19 exposure, shown as a Kaplan-Meier survival curve. Worker bees were sampled from all hives at week 4 and exposed or not exposed to Serratia marcescens kz19 under laboratory conditions for 10 days (*n* = 5 hives per condition, 3 cup cages per hive, at least 25 bees per cup cage). *, *P* < 0.05; ***, *P* < 0.001 (Cox proportional hazards model implemented in the package “survival”). (F) Glyphosate concentration detected in uncapped honey samples from control and treatment groups (*n* = 5 hives per group) at week 4.

For the first field experiment, 10 hives were randomly selected at site 1, divided into two groups, and exposed to either of the following conditions: 5 hives were treated weekly for 1 month with a single dose of 0.1% Roundup in sucrose syrup (0.5 liters), and the 5 other hives were treated with 0.5 liters sucrose syrup on the same treatment schedule (Fig. 3A; see Table S4 in the supplemental material). Sampling of bees began before initial treatment at week 0 (1 August 2018). At each sampling point, we compared microbial abundances and compositions between groups using 16S rRNA gene community profiling and qPCR analyses.

Although selected at random, bees from hives in the control group initially exhibited lower loads of total gut bacteria than did bees from hives in the treatment group (Fig. 3B), with fewer *Lactobacillus* Firm-4 and Firm-5 bacteria (week 0 in Fig. S4 in the supplemental material). Hives were treated immediately after sampling at week 0. One week after first exposure (week 1, 8 August 2018), bees were sampled again, and none of the differences previously observed remained (Fig. 3B; Fig. S4). However, significant decreases in *Snodgrassella alvi* and *Commensalibacter* abundances were observed in bees from Roundup-treated hives (Fig. 3C; Fig. S4). The same scheme of treatment was followed for the next 2 weeks, in which bees were first sampled and then treated each week. At weeks 3 (when the last treatment was provided to the hives, 22 August 2018) and 4 (1 week after finishing treatment, 29 August 2018), *Snodgrassella alvi* abundance remained significantly lower in bees from Roundup-treated hives (Fig. 3C). *Bifidobacterium* spp. also decreased in abundance in bees from these hives (Fig. 3D). One month after finishing treatment (week 7, 19 September 2018), the effects on the microbiota of bees from Roundup-treated hives not only persisted but also extended to most of the core bacterial species in the bee gut. We detected a significant decrease in abundance for total bacteria (Fig. 3B), with reduced loads of members of *Snodgrassella alvi* (Fig. 3C), *Bifidobacterium* (Fig. 3D), *Lactobacillus* Firm-5 (Fig. S4), *Gilliamella* (Fig. S4), and *Commensalibacter* (Fig. S4), and a significant increase in absolute abundance for members of *Bartonella* (Fig. S4) and *Frischella* (Fig. S4). We also checked the abundance for environmental bacteria in the bee gut. During the treatment, there was an increase in abundance for Lactobacillus kunkeei at week 3 and an increase in abundance for *Fructobacillus* spp. at weeks 3 and 4 (Fig. S4). These two bacterial groups are associated with floral nectar and are commonly found in honey bee hives (51).

**FIG 4:**
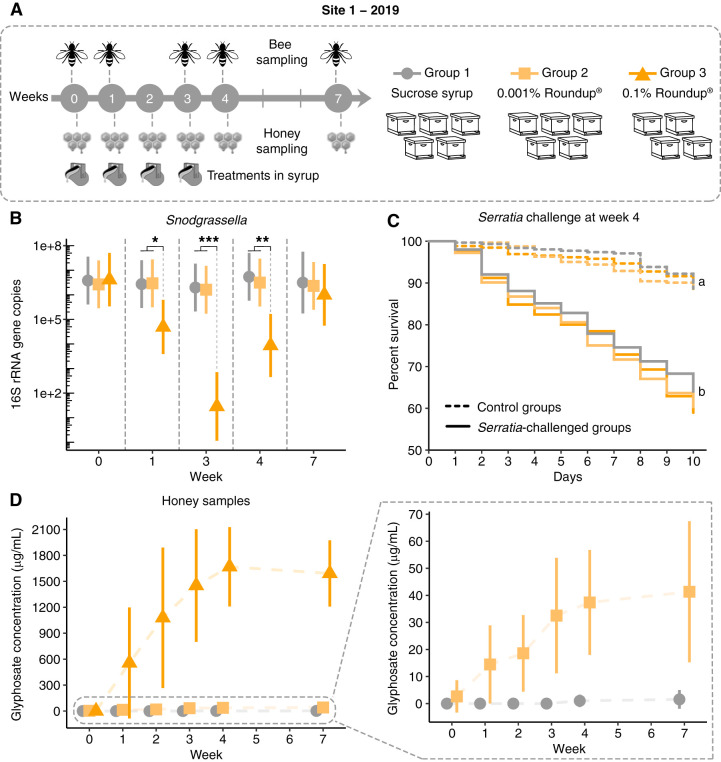
Gut microbial changes and susceptibility to bacterial infections in honey bees from hives exposed to Roundup formulation in site 1 in 2019. (A) Field experiment performed in site 1 in 2019. Fourteen hives were split into 3 groups to be exposed to 0.5 liters of sucrose syrup or 0.001% or 0.1% Roundup formulation dissolved in sucrose syrup at weeks 0, 1, 2, and 3. Treatment was placed in a reservoir inside the hives to avoid cross-contamination. Bees were sampled at weeks 0, 1, 3, 4, and 7. Uncapped honey samples were collected at weeks 0, 1, 2, 3, 4, and 7. (B) Scatterplot of *Snodgrassella alvi* abundance in the guts of bees sampled from control (sucrose syrup) and treatment (0.001% and 0.1% Roundup in syrup) groups on weeks 0, 1, 3, 4, and 7, with error bars indicating 95% confidence intervals. For group 1: *n* = 5 for weeks 0, 1, 3, and 4; *n* = 3 for week 7. For group 2: *n* = 5. For group 3: *n* = 4 for weeks 0 and 1; *n* = 3 for weeks 3, 4, and 7. Each hive is represented by 15 pooled bee guts. Generalized linear mixed-effects models assuming Poisson regression were used to compare changes in bacterial abundances between control and treatment hives per sampling time. Mixed models were fitted using the package lme4 (85) and followed by *post hoc* tests using the package emmeans (86). *, *P* < 0.05; **, *P* < 0.01; and ***, *P* < 0.001. (C) Survival rates of worker bees after Serratia marcescens kz19 exposure, shown as a Kaplan–Meier survival curve. Worker bees were sampled from representative hives from each group at week 4 and exposed or not exposed to Serratia marcescens kz19 under laboratory conditions for 10 days (*n* = 3 hives per condition, 3 cup cages per hive, at least 25 bees per cup cage). *, *P* < 0.05; ***, *P* < 0.001 (Cox proportional hazards model implemented in the package “survival”). (D) Glyphosate concentration detected in uncapped honey samples collected from control (*n* = 5, all weeks), 0.001% Roundup-treated (*n* = 5, all weeks), and 0.1% Roundup-treated (*n* = 4 for weeks 0, 1, and 2; *n* = 3 for weeks 3, 4, and 7) hives.

A second field experiment was performed in 2019 at two different sites, as an attempt to evaluate the effects of different concentrations and exposure levels of Roundup formulation delivered in two different matrices, sucrose syrup or water. At site 1, 14 hives were randomly selected to replicate the experiment performed in 2018 and to include a treatment group in which hives were exposed to a lower dose (0.001%) of Roundup formulation (Fig. 4A; Table S4). Treatment and sampling schemes were the same as those applied in the previous season. However, this time, guts were pooled to extract DNA and to perform 16S rRNA amplicon sequencing and qPCR analyses. The lower dose of Roundup formulation tested did not affect the abundance or the composition of the honey bee gut microbiota. On the other hand, the higher dose (0.1% Roundup) significantly decreased *Snodgrassella alvi* abundance during the treatment (weeks 1 and 3) and at 1 week after finishing treatment (week 4). This finding was similar to that in the experiment performed in 2018, but no effects were observed 1 month after finishing treatment (Fig. 4B). In 2019, we did not observe significant effects on other members of the gut community (see Fig. S5 in the supplemental material). Since we collected random bees from the hives, thus not controlling for age, pooling guts may have masked effects due to the potential presence of outliers, such as bees that were not exposed to the treatment.

**FIG 5:**
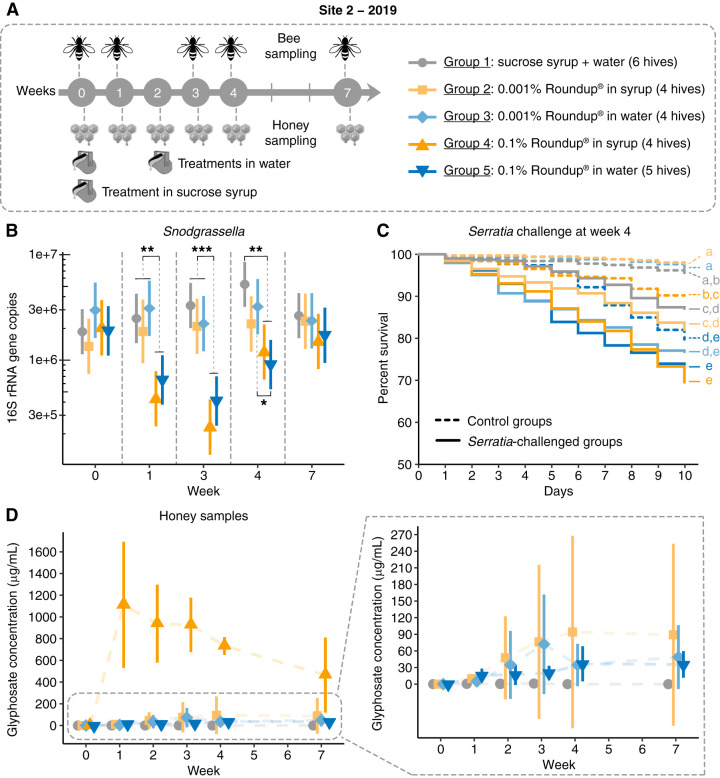
Gut microbial changes and susceptibility to bacterial infections in honey bees from hives exposed to Roundup formulation in site 2 in 2019. (A) Field experiment performed in site 2 in 2019. Twenty-three hives were split into 5 groups to be exposed to 0.001% or 0.1% Roundup formulation dissolved in sucrose syrup or water. Treatment in syrup (0.5 liters) was provided at week 0 in a reservoir inside each hive. Treatment in water (0.45 liters) was provided at weeks 0 and 2 in a glass bottle with a punched cap connected to a plastic boardman and attached to the hive entry. Bees were sampled at weeks 0, 1, 3, 4, and 7. Uncapped honey samples were collected at weeks 0, 1, 2, 3, 4, and 7. (B) Scatterplot of *Snodgrassella alvi* abundance in the guts of bees sampled from control and treatment groups on weeks 0, 1, 3, 4, and 7, with error bars indicating 95% confidence intervals. For group 1: *n* = 6 for weeks 0, 3, 4, and 7; *n* = 5 for week 1. For group 2: *n* = 4 for weeks 0, 3, 4, and 7; *n* = 3 for week 1. For groups 3 and 4: *n* = 4. For group 5: *n* = 5 for weeks 0, 1, 3, and 4; *n* = 4 for week 7 Each hive is represented by 15 pooled bee guts. Generalized linear mixed-effects models assuming Poisson regression were used to compare changes in bacterial abundances between control and treatment groups per sampling time. Mixed models were fitted using the package lme4 (85) and followed by *post hoc* tests using the package emmeans (86). *, *P* < 0.05; **, *P* < 0.01; and ***, *P* < 0.001. (C) Survival rates of worker bees after Serratia marcescens kz19 exposure, shown as a Kaplan-Meier survival curve. Worker bees were sampled from representative hives from each group at week 4 and exposed or not exposed to Serratia marcescens kz19 under laboratory conditions for 10 days (*n* = 3 hives per condition, 3 cup cages per hive, at least 25 bees per cup cage). *, *P* < 0.05; ***, *P* < 0.001 (Cox proportional hazards model implemented in the “survival” package in R). (D) Glyphosate concentration detected in uncapped honey samples collected from control (*n* = 6) and treatment (0.001% Roundup in syrup, *n* = 4; 0.001% Roundup in water, *n* = 4; 0.1% Roundup in syrup, *n* = 4; 0.1% Roundup in water, *n* = 5) groups at weeks 0, 1, 2, 3, 4, and 7.

At site 2, another 23 hives were randomly selected to be treated with similar concentrations of Roundup formulation as in site 1 (lower dose, 0.001%; or higher dose, 0.1%) in sucrose syrup (single dose at week 0), reducing exposure to one occurrence, or in water (single doses at weeks 0 and 2), changing the exposure matrix (Fig. 5A; Table S4). Single doses of 0.001% Roundup in sucrose syrup or in water did not significantly affect the abundance of the core bacterial members in the honey bee gut compared with that of the control group over a period of 2 months. On the other hand, *Snodgrassella alvi* abundance significantly decreased in the guts of bees treated with 0.1% Roundup at weeks 1, 3, and 4, regardless of exposure matrix, syrup or water (Fig. 5B). Other changes were observed, such as increases in the abundance of *Lactobacillus* Firm-4 (week 1) and *Bartonella* (week 7) in bees treated with 0.1% Roundup in water (see Fig. S6 in the supplemental material). There was also an increase in the abundance of environmental bacteria, such as species of *Enterobacteriaceae*, in the guts of bees treated with the formulation at week 3 (i.e., 3 weeks after treatment was provided) (Fig. S6).

**(ii) Effects on susceptibility to *Serratia* infection.** Bacterial challenge assays were performed under laboratory conditions with bees collected at week 4 of each field experiment performed in 2018 and 2019. In 2018, bees collected from hives treated with 0.1% Roundup in syrup and exposed to the opportunistic bacterial pathogen *Serratia marcescens* exhibited increased mortality compared with bees collected from control hives exposed or not exposed to *Serratia marcescens* or bees from hives treated with 0.1% Roundup in syrup but not exposed to *Serratia marcescens* (Fig. 3E). We did not observe the same pattern in 2019 for bees sampled from site 1; both control and treatment groups exhibited similar mortality rates when exposed to *Serratia marcescens* but exhibited higher rates than those not exposed to *Serratia marcescens* (Fig. 4C).

Effects observed at site 2 in 2019 were more similar to those observed in 2018. This time, bees collected from hives treated with a single dose of 0.1% Roundup in syrup or water and exposed to *Serratia marcescens* exhibited increased mortality to that observed for bees collected from control hives exposed to *Serratia marcescens* (Fig. 5C). This effect was not observed for bees collected from hives treated with 0.001% Roundup in syrup or water and exposed to *Serratia marcescens* (Fig. 5C). To determine whether this increased mortality was attributable to the effects of the formulation on the gut microbiota or to direct effects on bees, we included control groups not exposed to *Serratia marcescens*. In bees collected from hives treated with the formulation, but not exposed to *Serratia marcescens*, survival rates were only significantly affected by the formulation when bees were treated with 0.1% Roundup in water (Fig. 5C), suggesting that direct and indirect effects of the formulation on bees may play a role in the increased susceptibility to *Serratia marcescens*, depending on the route of exposure.

### (iii) Glyphosate transference to hive compartments.

During the first field experiments in 2018, we observed that treatments given to the colonies were being depleted overnight. Therefore, we investigated whether the bees were consuming the entire treatment solution or storing part of it in hive combs. One week after the last treatment, on week 4, honey from uncapped hive combs was sampled and processed to detect glyphosate by high-resolution liquid chromatography-mass spectrometry (LC-MS). Interestingly, glyphosate was detected in samples collected from all treatment hives in the range of 800 to 1600 μg/ml but not in control hives (Fig. 3F).

Because of this intriguing fact, in 2019, we decided to collect uncapped honey from the colonies throughout the experiments in both sites 1 and 2. As expected, at week 0, before the beginning of treatments, glyphosate was not detected in uncapped honey collected from the hives (Fig. 4D and Fig. 5D). However, after week 1, all the treatment hives in site 1 contained increasing concentrations of glyphosate, which were at the same magnitude of the doses provided in site 1 (0.001% or 0.1% glyphosate in herbicide formulation), and glyphosate remained in the hives even 1 month after finishing exposure (Fig. 4D).

A similar trend was observed at site 2 for the hives treated with sucrose syrup containing the Roundup formulation. However, this time the initial peak in concentration decreased with time but still persisted even 2 months after finishing a single treatment (Fig. 5D). Hives treated with the formulation in water also contained contaminated uncapped honey, but concentrations were lower than the ones detected for hives treated with 0.1% Roundup in syrup. Glyphosate concentrations in uncapped honey were similar in hives treated with either 0.001% or 0.1% Roundup in water, and contamination persisted even several weeks after treatment. This finding suggests that bees can also be exposed to glyphosate in contaminated water sources near agricultural sites. The small amount of glyphosate detected in hives suggests that most of the water was consumed or used by the bees, although part of it evaporated (see Fig. S7 in the supplemental material). Interestingly, very low concentrations of glyphosate were detected in a few control hives after treatment, suggesting cross-contamination between groups carried by the bees.

### Topical exposure of honey bees to glyphosate in herbicide formulation—effects on survival rates and gut microbial composition.

**FIG 6:**
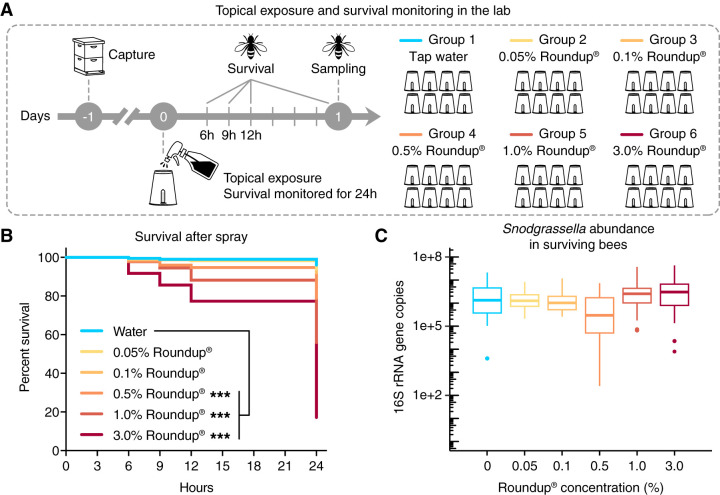
Survival rates and gut microbial changes of honey bees topically exposed to Roundup formulation under laboratory conditions. (A) Worker bees were split into 6 groups to be sprayed with different concentrations of glyphosate in the herbicide formulation in water. Survivorship was monitored for 24 hours under laboratory conditions. (B) Survival rates of worker bees after topical exposure to a glyphosate-based formulation over a period of 24 hours, shown as a Kaplan-Meier survival curve (*n* = 10 cup cages per group, 38 to 40 bees per cup cage). ***, *P* < 0.001 (Cox proportional hazards model implemented in the package “survival”). (C) Box plots of *Snodgrassella alvi* abundance in the guts of survived bees 24 hours after spray, measured by qPCR (*n* = 15 bees per group). Box-and-whisker plots show high, low, and median values, with lower and upper edges of each box denoting first and third quartiles. No significant changes were observed by Kruskal-Wallis test.

A worst-case scenario would occur when bees are sprayed directly and thus topically exposed to high concentrations of glyphosate-based formulations at the time of application. Therefore, we investigated the topical effects of Roundup on the health and the gut microbiota of honey bees. For that, we tested different concentrations of the formulation, ranging from 0.05% to 3% Roundup in water, and monitored the effects on bees and on the gut microbiota under both laboratory and hive conditions. After spray exposure of bees under laboratory conditions, survival was monitored for the next 24 h (Fig. 6A). Significant increases in mortality were observed for bees sprayed with 0.5%, 1.0%, or 3.0% Roundup compared with bees sprayed with water (Fig. 6B). This increased mortality was observed for the 3.0% Roundup-exposed group in all of the survival monitoring times (6, 9, 12, and 24 h after topical exposure), whereas the effects on the 0.5% and 1.0% Roundup-exposed groups were only apparent 12 h after topical exposure (see Fig. S8 in the supplemental material). A dose response of bee survival to topical exposure was also observed, with a 50% effective dose (ED_50_) value, i.e., the half maximal effective concentration of formulation at which bee survival is reduced by 50%, of 1.25% ± 0.38% glyphosate in herbicide formulation (see Fig. S9 in the supplemental material). In initial trials, bees sprayed with 1.0% glyphosate in water did not die more than bees sprayed with only water, even 24 h after exposure, suggesting that other components of the formulation are responsible for the increased mortality of these topically exposed bees (see Fig. S10 in the supplemental material; see Table S5 in the supplemental material).

**FIG 7:**
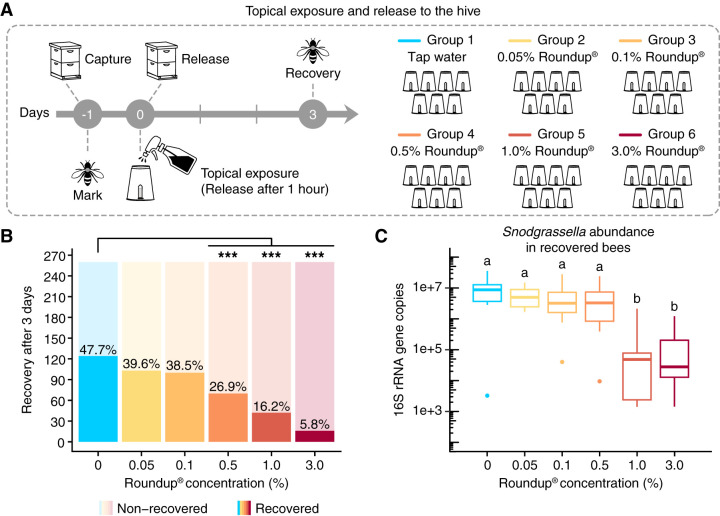
Recovery rates and gut microbial changes of honey bees topically exposed to Roundup formulation under hive conditions. (A) Worker bees were split into 6 groups and marked on the thorax with different colors of paint. Groups were sprayed with different concentrations of a glyphosate-based formulation in water and released back to their hive. All marked bees were recovered on day 3 postspray. (B) Recovery rates of worker bees topically exposed to a glyphosate-based formulation on day 3 postspray (*n* = 7 replicates per group, 35 to 40 bees per replicate). ***, *P* < 0.001 (chi-squared test followed by Bonferroni’s correction). (C) Box plots of *Snodgrassella alvi* abundance in the guts of recovered bees on day 3 postspray, measured by qPCR (*n* = 15 bees per group). Box-and-whisker plots show high, low, and median values, with lower and upper edges of each box denoting first and third quartiles. Groups with distinct letters are statistically different (*P* < 0.001, Kruskal-Wallis test followed by Dunn’s multiple-comparison test).

After topically exposed bees were released back to the hive, recovery rates were measured at day 3 postexposure. Compared with control bees, which were sprayed with water, significant reductions in recovery rates were observed for the groups sprayed with 0.5%, 1.0%, and 3.0% Roundup in water (Fig. 7; see Table S6 in the supplemental material). We repeated this experiment for specific concentrations (0.1%, 1.0%, and 3.0% Roundup) and found similar results. For the replicate experiments, each concentration was evaluated individually along with a control group (see Fig. S11 in the supplemental material).

Surviving bees (from the laboratory experiment) and recovered bees (from the hive experiment) were sampled to investigate whether the gut microbiota was affected by topical exposure to Roundup formulation. For this investigation, we checked *Snodgrassella alvi* abundance by qPCR and used it as an indicator of microbiota perturbation. Interestingly, *Snodgrassella alvi* abundance was reduced in the guts of recovered bees sprayed with 1.0% or 3.0% Roundup (Fig. 7C), but no significant effects were observed for bees kept under laboratory conditions (Fig. 6C). This finding suggests that Roundup formulation in water, besides reducing survivorship, can also affect the gut microbiota of topically exposed honey bees.

## DISCUSSION

A main impact of herbicide use on ecosystems, including bees and other invertebrates, is the loss of wild plants that provide food and shelter (52, 53). But impacts on susceptible organisms other than plants can also occur. Recent studies have demonstrated that glyphosate, an herbicide with bacteriostatic properties, can affect the microbiota of animals (12, 48, 49, 54–59). The consequences of such microbial perturbations for hosts are still not fully understood and likely vary depending on the roles of the microbiota in host health. Honey bees, for example, have coevolved with a specific gut bacterial community (60), with benefits ranging from metabolic contributions (35, 36, 38) to host immune system stimulation (34, 39). Dysbiosis has negative consequences for bee health, such as increased susceptibility to infection by pathogens (61, 62).

It was previously shown that glyphosate, under laboratory conditions, perturbs the honey bee gut microbiota by reducing the abundance of *Snodgrassella alvi* (12, 48), a primary bacterial colonizer that forms a biofilm on the ileum wall that probably facilitates the establishment of secondary colonizers, such as members of *Gilliamella*, *Bifidobacterium*, and *Lactobacillus*. Experiments that demonstrated these effects were performed with pure glyphosate. However, glyphosate is commonly found in herbicide formulations in the form of a salt mixed with surfactants to increase water solubility and penetration of the targeted organisms. Thus, the effects of glyphosate in herbicide formulations may differ from the effects of pure glyphosate (63).

Here, we demonstrate that glyphosate in herbicide formulation also affects the gut microbiota of honey bees, regardless of route (oral or topical) or source (sucrose syrup or water) of exposure. Under field conditions, a single oral exposure to the herbicide formulation was enough to reduce the abundance of *Snodgrassella alvi* in the bee gut. This effect persisted in subsequent weeks during the experiments, regardless of whether further exposures occurred. In some trials, the effects of the formulation extended to other beneficial bacteria, such as *Bifidobacterium* spp., and persisted even 1 month after treatments ended. This is probably due to glyphosate, and possibly other components of the formulation, accumulating in parts of the hive environment, such as in honeycombs, which would prolong the time of exposure. The decrease in the abundance of gut-restricted bacteria led to an increase in environmental bacteria, such as *Fructobacillus* spp. and Lactobacillus kunkeei, two prolific bacterial groups found in nectar and in honey bee hives (51).

These microbial perturbations were not linked to any apparent negative effect on colony fitness. Indeed, microbiota perturbation due to glyphosate exposure is not expected to produce an immediate, obvious increase in bee death (40), but instead more subtle effects of nutritional stress or increased susceptibility to bacterial infection (12). Because sick honey bee workers are excluded from hives to prevent the spread of disease to nestmates (64), dead bees are unlikely to accumulate in the hive. Therefore, we investigated the impacts of glyphosate-based formulation on bees by performing infection and mark-recapture experiments. Infection experiments were done with bees collected from hives used in the field experiments after herbicide exposure. We challenged these bees with Serratia marcescens, an opportunistic bacterium that can cause disease in adult bees (3, 4). This experiment was done in the laboratory to avoid the spread of pathogens through the apiary. Under these conditions, we observed reduced survival rates for bees collected from hives exposed to the formulation and challenged with Serratia marcescens in two of three trials. Moreover, we observed reduced recovery rates for bees orally exposed to the formulation in three of five mark-recapture experiments using bees from a different apiary. Together, these experiments suggest that honey bees are affected by the glyphosate-based formulation, but effects vary based on colony status.

Bees can also be topically exposed to glyphosate-based herbicides during application, and concentrations under these circumstances are very high. Here, we show that honey bees sprayed with different concentrations of the glyphosate-based formulation exhibit increased mortality compared with bees sprayed with pure glyphosate or water. This finding suggests that surfactants or other unknown adjuvants in the formulation are responsible for the increased mortality, as observed in other studies (65, 66). We performed experiments under both laboratory and hive conditions, and survival rates were dose-dependent and similar among these experiments. Surprisingly, topical exposure to the formulation also affected the gut microbiota of honey bees, showing that topically exposed, surviving bees will have a defective gut microbiota and may become more susceptible to other environmental stressors, such as opportunistic bacterial pathogens.

The effects of glyphosate and its formulations have been tested for other microbial communities, both *in vitro* and *in vivo*. Common beneficial gut bacteria of animals, including members of *Bifidobacterium*, *Enterococcus*, and *Lactobacillus*, were more susceptible to a glyphosate-based herbicide *in vitro* than some pathogens, such as members of *Clostridium* and *Salmonella* (58), but this was not observed in another study (67). Studies of rats have shown that glyphosate-based herbicides can affect behavior (54), as well as the gut microbiome (54, 57, 68). Other studies have suggested that sufficient dietary supplementation of aromatic amino acids counters the negative effects of glyphosate or glyphosate-based formulations *in vitro* in representative bacteria of the human gut microbiome and also *in vivo* in rat gut microbiota (56). Another study (57) tested effects of chronic exposure at environmental concentrations of glyphosate (0.1 ppb) and found negative effects on the gut microbiota. The impacts on microbiota may be stronger in cases of poor nutrition, especially if the availability of aromatic amino acids is low.

The EPSPS enzyme varies among bacterial species in presence and in tolerance to glyphosate. Among core members of the bee gut, *Snodgrassella alvi*, *Bifidobacterium* spp., and *Gilliamella* spp. contain a functional shikimate pathway and can produce their own aromatic amino acids. Exposure of bees to glyphosate directly inhibits the growth of *Snodgrassella alvi*, *Bifidobacterium* spp., and sometimes *Gilliamella* spp. This inhibition may in turn decrease the abundance of aromatic amino acids, and depress the growth of bacteria lacking EPSPS, such as *Lactobacillus* spp., which rely on the uptake of aromatic amino acids produced by other community members. We observed this correlation not only in this study but also in previous experiments (12). Most of the nutrients that bees acquire from pollen and nectar are absorbed in the proximal region of the gut (midgut), and only hard-to-digest components of the pollen cell wall pass to the distal region of the bee gut (ileum and rectum), where the microbial community resides (69–71). A mutagenesis study of *Snodgrassella alvi* (72) showed that genes for the production of aromatic amino acids (including the gene encoding EPSPS) were required for bacterial survival in the bee gut. Metabolomic analyses of different bee gut compartments demonstrate higher levels of the aromatic amino acids tryptophan and tyrosine in the ileum and rectum of bees with a normal microbiota than those in microbiota-free bees, whereas midgut concentrations of aromatic amino acids are similar in both groups (38).

These experimental observations and predictions assume that the primary effect of glyphosate is inhibition of the EPSPS enzyme. Indeed, glyphosate resistance in many genetically modified organisms is based on the expression of a class II EPSPS from Agrobacterium tumefaciens (73). However, glyphosate not only can inhibit class I EPSPS enzymes but can also act as a potent chelator for micronutrients that are essential cofactors of enzymes and help in the stabilization of proteins in most organisms (74). Glyphosate binds divalent cations, such as Mg^2+^, Ca^2+^, Fe^2+^, Mn^2+^, Cu^2+^, and Zn^2+^, and forms stable complexes. Moreover, some gut-associated *Lactobacillus* species require large amounts of Mn for protection from oxidative damage (75, 76) and thus could be inhibited if Mn is chelated by glyphosate. A reduction in the bioavailability of cations could compromise organism growth in a way similar to the depletion of aromatic amino acids.

Glyphosate exposure does not immediately kill plants, which can persist as a source of contaminated food for bees for at least 5 days after spraying (40); glyphosate-resistant weeds and crops may provide contaminated nectar and pollen for even longer. Bees can also be exposed to the herbicide when collecting water from ponds and ditches or plant resin to make propolis. Since foraging bees bring nectar, pollen, and propolis materials back to the colony, exposure can be extended to young worker bees. For example, glyphosate was quantified in pollen (100 to 600 mg/kg) and nectar (10 to 30 mg/kg) collected by foraging bees in a semifield experiment (40). Researchers also fed hives with glyphosate, but not the formulation, dissolved in sucrose syrup but did not find effects on bee mortality (40).

In our field experiments, we also detected a transfer of glyphosate from bees to hive compartments. Uncapped honey exhibited glyphosate concentrations in the same magnitude of concentration provided in the sucrose syrup treatment. This probably happened because treatments were provided inside the hive, instead of spraying nearby plants, to avoid cross-contamination. Unfortunately, this precludes any comparisons between the glyphosate concentrations detected in our experiments with concentrations detected in honey in other studies (44–47), but it does show that bees can directly transfer contaminated resources to hive compartments. A recent study using bees under laboratory conditions showed that glyphosate has a persistent impact on the gut microbiota of young adult workers whether exposure occurs during or after microbiota establishment and that this impact is dose dependent (88).

Therefore, several variables could dictate the magnitude of effects of glyphosate or herbicide formulations on honey bees and their gut microbiota. From the herbicide side, level and route of exposure are the main factors. From the bee side, variation in colony health and population densities, exposure to different pathogens and parasites, and differences in nutritional status due to food resource availability and seasonal variations are likely key.

## MATERIALS AND METHODS

### Chemicals and solutions.

The glyphosate standard, in the acid form, was purchased from Research Products International, USA (lot 32612-38399). The Roundup ProMax formulation (a copyrighted product of the Monsanto Company) was purchased from an agricultural retailer; this formulation contains 48.7% (wt/vol) or 660 g/liter of potassium salt of glyphosate, equivalent to 540 g/liter of the acid glyphosate.

For the oral exposure experiments, the glyphosate standard was initially dissolved in distilled water and then diluted to the final concentration with filter-sterilized 0.5 M sucrose syrup, whereas the formulation was directly diluted in filter-sterilized 0.5 M sucrose syrup. For the topical exposure experiments, both the standard and the formulation were directly diluted in tap water. The final concentrations used in the experiments were achieved by considering the initial concentration of glyphosate acid in the formulation.

### Honey bee rearing.

For the laboratory and hive recovery experiments, honey bees (the European Apis mellifera) were obtained from outside hives kept on the rooftop of J. T. Patterson Laboratories Building at UT-Austin (latitude, 30.287913; longitude, −97.736183). These hives are assigned a unique number and name, which usually change when requeening is required. For the field experiments, honey bees were obtained from hives kept on two different sites at Driftwood, TX (site 1 at latitude, 30.1114998 and longitude, −98.0212251; site 2 at latitude, 30.115057 and longitude, −98.0249667). These hives did not exhibit signs of brood or adult bee diseases during the experiment. The surrounding environment is rural and is not actively farmed, so we expected no glyphosate exposure from other sources.

In the field experiments, we provided weekly single doses of 0.5 liters of a 1:1 (wt/vol) sucrose solution to the hives in site 1 (August 2018 and 2019) and a single dose of the same sucrose solution to the hives in site 2 (August 2019). Based on references 40 and 77, a small hive would require 135 g/day of nectar and 4.5 g/day of pollen for normal development. We provided 500 g sugar (assuming 50% sugar content in the sucrose solution), which should be consumed in 3 to 4 days and would probably reduce foraging activity during that time.

### Oral exposure experiments.

**(i) Laboratory conditions.** A brood frame was obtained from a honey bee hive kept at UT-Austin, transferred to a frame cage, and placed in an incubator at 35°C and ∼60% relative humidity to simulate hive conditions until adults emerged. Newly emerged bees were transferred to cup cages and divided into three groups, which were fed (i) sterile sucrose syrup, (ii) 1.0 mM glyphosate dissolved in sterile sucrose syrup, or (iii) 1.0 mM glyphosate in herbicide formulation (or Roundup) dissolved in sterile sucrose syrup. All groups were allowed to acquire their normal microbiota during treatment by addition of a suspension of freshly prepared gut homogenate from hive bees to the bee bread, as described in previous studies (12, 33). After 5 days of treatment, 8 bees from each group were sampled and stored at –80°C.

**(ii) Hive recovery.** Hundreds of worker bees were collected from inside hive number 8 (Imperial) in fall 2018, immobilized at 4°C, and split into two groups, control and treatment, which were marked with white and pink paint on the thorax, respectively. Then, bees were transferred to cup cages in groups of 40 bees, with 10 replicates per condition. The control group was fed sterile sucrose syrup, while the treatment group was fed 0.1% Roundup in sterile sucrose syrup. During treatment, bees were maintained in an incubator (at 35°C and ∼60% relative humidity) and mortality was censused on a daily basis. After 3 days of treatment, bees from each group were quickly immobilized with CO_2_ and pooled in plastic containers. Fifteen bees were randomly sampled from each group and stored at –80°C. This was considered day 0 posttreatment. A total of 348 bees from each group were returned to the original hive by placing them in the top hive box (containing no frames). Three days after reintroduction to the hive, or day 3 posttreatment, marked bees were temporarily recaptured to be counted. For that recapture, every frame of the hive was repeatedly inspected until no more marked bees could be found. Fifteen bees were sampled from each group, and those remaining were released back to the hive to be sampled again at day 5 posttreatment. All sampled bees were placed in Falcon tubes and stored at –80°C. This experiment was repeated 4 more times in different seasons, using bees from different hives, and swapping group colors as described in Table S3. For treatment, release, and recovery steps, we followed the same protocol described for the first experiment, but bees were only sampled at days 0 and 3 posttreatment. Moreover, a color marking validation experiment was conducted with worker bees from hive number 9 (Alsatian); in this case, both groups were fed sterile sucrose syrup (Table S3).

### Topical exposure experiments.

**(i) Laboratory conditions.** For experiments 1 and 2, hundreds of worker bees were taken from inside hive number 3 (Firefly) in spring 2019, immobilized at 4°C, and then transferred to cup cages in groups of 40 bees, with a total of 24 cup cages. Bees were maintained in an incubator at 35°C and ∼60% relative humidity, and they were provided with sterile sucrose syrup in tubes attached to the top of the cup cage. The following day, filter paper lining, sterile sucrose syrup tubes, and dead bees were removed from cup cages, and the remaining bees were sprayed from the top of the cup cage with ∼1.2 ml of either tap water or 1.0% Roundup in tap water (12 cup cages, or 436 bees per group). Mortality rates were censused 6 hours after topical exposure. This experiment was replicated in summer 2019, but with a total of 10 cup cages, or 385 bees per group.

For experiments 3 and 4, hundreds of worker bees were taken from inside hive number 3 (Firefly) in summer 2019, immobilized at 4°C, and then transferred to cup cages in groups of 40 bees, with a total of 20 cup cages. Bees were maintained in an incubator at 35°C and ∼60% relative humidity, and they were provided with sterile sucrose syrup in tubes attached to the top of the cup cage. The following day, sucrose syrup tubes and dead bees were removed from cup cages, and the remaining bees were split into 4 groups which were sprayed from the top of the cup cage with ∼1.2 ml of either tap water, 0.1% Roundup in tap water, 1.0% Roundup in tap water, or 1.0% glyphosate in tap water (10 cup cages, or 386 to 388 bees per group). Mortality rates were censused at 6 h and 24 h after topical exposure. This experiment was replicated in the same season using bees from hive number 0 (Avocado), with 7 cup cages or 266 bees per group, and mortality rates were censused at 6 h, 9 h, 12 h, and 24 h after topical exposure.

For experiments 5 and 6, hundreds of worker bees were taken from inside hive number 1 (Leviathan) in fall 2019, placed in a cold room until immobilized, and then transferred to cup cages in groups of 40 bees, with a total of 48 cup cages. Bees were maintained in an incubator at 35°C and ∼60% relative humidity, and they were provided with sterile sucrose syrup in tubes attached to the base of the cup cage. The following day, dead bees were removed, and the cup cages were split into 6 groups which were sprayed with ∼1.2 ml of either tap water; or 0.05%, 0.1%, 0.5%, 1.0%, or 3.0% Roundup in tap water (8 cup cages, or 310 bees per group). Mortality rates were censused at 6 h, 9 h, 12 h, and 24 h after topical exposure. This experiment was replicated in the same season using bees from hive number 6 (Pyrenees), with 10 cup cages, or 397 bees per group.

These topical exposure experiments performed under laboratory conditions are summarized in Table S5.

**(ii) Hive recovery.** For experiments 1, 2, 3, and 4, hundreds of worker bees were collected at four different times from inside hive number 2 (Newfoundland) in fall 2019 and placed at 4°C. After approximately 3 hours, immobilized bees were marked on their thorax with their respective group colors, either green or blue, for control or treatment, respectively. These bees were placed into cup cages in groups of 40 bees with 10 replicates per group, provided sucrose syrup in tubes attached to the base of the cup cage, and put into an incubator at 35°C and ∼60% humidity. The next day, dead bees were removed, and the remaining ones were briefly immobilized with CO_2_ and sprayed from the top of the cup cage with ∼1.2 ml of either tap water or Roundup formulation dissolved in tap water (0.1%, 1.0%, 1.0%, and 3.0% formulation for experiments 1, 2, 3, and 4, respectively). After 1 hour of topical exposure, bees were released in front of hive number 2. After 3 days, marked bees were recovered.

For experiment 5, hundreds of worker bees were collected one single time from inside hive number 6 (Pyrenees) in fall 2019 and placed at 4°C. After approximately 3 hours, immobilized bees were marked on their thorax with their respective group colors (white, green, orange, pink, yellow, or blue) for control and different treatments. These bees were placed into cup cages in groups of 40 bees with 7 replicates per group, provided sucrose syrup in tubes attached to the base of the cup cage, and put into an incubator at 35°C and ∼60% humidity. The next day, dead bees were removed, and the remaining ones were briefly immobilized with CO_2_ and sprayed from the top of the cup cage with ∼1.2 ml of either tap water or Roundup formulation dissolved in tap water (0.05%, 0.1%, 0.5%, 1.0%, and 3.0% formulation). After 1 hour of topical exposure, bees were released in front of hive number 6. After 3 days, marked bees were recovered.

These topical exposure experiments followed by hive recovery are summarized in Table S6.

### Field experiments.

Several honey bee hives were transferred to two different sites on privately owned land in Driftwood, TX, approximately 2 months before the beginning of experiments in 2018 and 2019. Hives were comprised of 8 frames and approximately 8,000 to 10,000 worker bees, which were allowed to forage freely. To the best of our knowledge, there were no nearby flowering crops and few flowering wild plants, probably due to the dry season that preceded the experiments in 2018 (see Fig. S12 in the supplemental material). However, flowering plants were more abundant in 2019, probably due to the rainy season that preceded the experiments in 2019 (Fig. S12). Moreover, colonies were provided with a limited number of food resources to encourage subsequent feeding. All colonies were generally assessed during the period of the experiment, and no behavioral or physical abnormalities were observed.

The first field experiment was performed at site 1 in August/September 2018. Five hives were selected to be part of the control group and were supplemented with 0.5 liters of sucrose syrup, whereas another 5 hives were selected to be part of the treatment group and were exposed to 0.5 liters of 0.1% Roundup dissolved in sucrose syrup. Honey bees were sampled from each hive in the beginning of the experiment, at week 0, after which treatments were added in containers allocated inside each hive to avoid cross-contamination. Hives were inspected on a weekly basis during which bees were sampled before adding fresh treatments on weeks 1, 2, and 3. Honey bees were also sampled on weeks 4 (1 week after final treatment) and 7 (1 month after final treatment). Fifteen bees from each hive per sampling time were used to extract DNA, totaling 750 samples.

The second field experiment was performed at sites 1 and 2 in August/September 2019. In site 1, hives were supplemented with 0.5 liters of sucrose syrup (control; *n* = 5), 0.001% Roundup in sucrose syrup (0.001R-S, *n* = 5), or 0.1% Roundup in sucrose syrup (0.1R-S, *n* = 4); following the same treatment and sampling schemes as in the first field experiment. One hive from the 0.1R-S treatment group died on week 2. Fifteen bees from each hive per sampling time were dissected, and guts were pooled according to hive for DNA extraction, totaling 67 samples.

In site 2, hives were split into 5 groups. Hives from the control group (*n* = 6) were supplemented with 0.5 liters of sucrose syrup and 0.45 liters of tap water, whereas hives from the treatment groups were supplemented with 0.001% or 0.1% Roundup dissolved in sucrose syrup (0.001R-S, *n* = 4; and 0.1R-S; *n* = 4, respectively) or in water (0.001R-W, *n* = 4; and 0.1R-W, *n* = 5, respectively). Hives were supplemented with a single dose of sucrose syrup at week 0, as well as with water at weeks 0 and 2. Sucrose syrup was added in containers allocated inside each hive, whereas water was added to a glass bottle with a punched cap connected to a plastic boardman and attached to the hive entry. Honey bees and uncapped honey were sampled directly before adding treatments to the hives (week 0) and at 1, 2, 3, 4, and 7 weeks after providing treatments to the hives. Fifteen bees from each hive per sampling time (except for week 2) were dissected, and guts were pooled respective to hive for DNA extraction, totaling 115 samples.

### *Serratia* infection experiments.

*Serratia* infection experiments were performed with worker bees collected from hives used in the field experiments (site 1 in 2018 and sites 1 and 2 in 2019). For each experiment, approximately 200 worker bees were collected from each selected hive at week 4 (at least three hives per group) and brought back to the laboratory. Then, they were briefly immobilized at 4°C and transferred to cup cages in groups of at least 25 bees, with 6 replicates per group. Cup cages were transferred to growth chambers simulating hive conditions. The next day, each group was divided into two subgroups; one subgroup was used as a control and provided only sterile sucrose syrup, whereas the other group was challenged with the opportunistic pathogen Serratia marcescens strain kz19. For that challenge, a Serratia marcescens kz19 suspension in sucrose syrup with an optical density (OD) of 0.5 was provided to the cup cages in feeding tubes. Briefly, Serratia marcescens kz19 bacteria were grown in LB broth at 37°C the night before the experiment. The OD at 600 nm (OD_600_) was measured, and cells were washed with phosphate-buffered saline (PBS) and diluted to an concentration of OD of 0.5 in proportions of 1 to 4 of PBS and sucrose syrup, respectively. The bacterial suspension was administered in feeding tubes. In each experiment, survivorship was monitored and recorded each day for 10 days. Kaplan-Meier survival curves were generated in GraphPad Prism.

### DNA extraction, qPCR analysis, and 16S rRNA library preparation.

For the laboratory and hive experiments, sampled honey bees were placed in sterile Falcon tubes and, while still alive, were transferred to a freezer at –80°C. For the field experiments, sampled honey bees were placed in clean tubes and immediately flash frozen on site in a dry ice and ethanol mixture until transferred to a freezer at –80°C.

For the laboratory, hive recovery, and 2018 field experiments, DNA was extracted from individual guts, following the protocol previously described (60). For the 2019 field experiments, DNA was extracted from pooled guts (15 dissected guts per hive were pooled) following the same protocol with the following modifications: dual extraction with 0.75 ml phenol:chloroform:isoamyl (25:25:1), dual cleaning with 1.0 ml of cold 75% ethanol, and resuspension of DNA pellet in 200 μl water.

All DNA samples were 10-fold diluted to be used as the template for qPCR analyses, as described in reference 12, and for 16S rRNA library preparation.

Library preparation consisted of two PCR steps. PCR 1 was designed to amplify the V4 region of the 16S small subunit (SSU) rRNA gene and was performed in 20-μl triplicate reactions using 515F (5′-TCGTCGGCAGCGTCAGATGTGTATAAGAGACAGGTGYCAGCMGCCGCGGTA-3′) and 806R (5′-GTCTCGTGGGCTCGGAGATGTGTATAAGAGACAGGGACTACHVGGGTWTCTAAT-3′) primers (both at a 200 nM final concentration) and 5 Prime HotMasterMix (2.5×; Quantabio, MA, USA). Cycling conditions consisted of 94°C for 3 min; 30 cycles of 94°C for 45 s, 50°C for 60 s, and 72°C for 90 s; and then 72°C for 10 min. PCR 1 products were combined, purified with 0.8× HighPrep PCR magnetic beads (Magbio, MD, USA), and diluted to a final volume of 52.5 μl. PCR 2 was designed to attach dual indices and Illumina sequencing adapters to the PCR 1 products and was performed in 25-μl single reactions using a unique combination of N7XX (5′-CAAGCAGAAGACGGCATACGAGATNNNNNNGTCTCGTGGGCTCGG-3′) and S5XX (5′-AATGATACGGCGACCACCGAGATCTACACNNNNNNTCGTCGGCAGCGTC-3′) index primers (both at a 400 nM final concentration) and 5 Prime HotMasterMix (2.5×, Quantabio). Cycling conditions consisted of 94°C for 3 min; 10 cycles of 94°C for 20 s, 55°C for 15 s, and 72°C for 60 s; and then 72°C for 10 min. PCR 2 products were purified with 0.8× HighPrep PCR magnetic beads (Magbio), diluted to a final volume of 27.5 μl, and quantified fluorometrically (Qubit; Thermo Fisher Scientific Inc.). Samples (50 ng each) were split into four pooled libraries. The first pooled library consisted of samples from weeks 1 and 3 of the field experiment performed in 2018 (total of 300 samples). The second pooled library consisted of samples from weeks 0 and 7 of the field experiment performed in 2018 (total of 300 samples). The third pooled library consisted of 150 samples from week 4 of the field experiment performed in 2018, 90 samples from the first oral exposure, hive recovery experiment, and 24 samples from the oral exposure, laboratory experiment. The fourth pooled library consisted of 182 samples from the field experiments performed in 2019. Each library was loaded onto an Illumina iSeq cartridge according to the manufacturer’s instructions and subjected to Illumina sequencing on the iSeq platform (2 × 150-bp sequencing run; instrument model number FS10000184); 5% PhiX was used to check the quality of the runs.

### Processing of 16S rRNA amplicon data.

Illumina sequence reads were demultiplexed on the basis of the barcode sequences by the iSeq software and then processed according to experiment in QIIME 2 version 2019.10 (78). Due to the lack of sufficient overlap between forward and reverse reads, downstream analyses were performed with forward reads only. Primer sequences were removed using the cutadapt plugin (79). Then, reads were truncated to 120 bp, filtered, and denoised; and chimeric reads were removed using the DADA2 plugin (80). Taxonomy was assigned to amplicon sequence variants (ASVs) using the SILVA database in the feature-classifier plugin (81). Reads with lower than 0.1% abundance were removed from the data set using the feature-table plugin, as well as unassigned, mitochondrial and chloroplast reads using the taxa filter-table plugin. The absolute abundance for each bacterial species was estimated by multiplying the total number of 16S rRNA gene copies obtained by qPCR by the percent relative abundance of each species, adjusting based on genomic 16S rRNA gene copy number, as in reference 62.

### Quantification of glyphosate in honey samples.

Approximately 2-ml samples of uncapped honey were collected from hives at site 1 in 2018 (week 4) and at sites 1 and 2 in 2019 (weeks 0, 1, 2, 3, 4 and 7). These samples were preserved at –20°C until submitted to an extraction protocol to detect and quantify glyphosate. Briefly, 1.00 ± 0.01 g of honey was weighed in a 50-ml Falcon tube and homogenized with 4.3 ml of a solution of 50 mM acetic acid and 10 mM Na_2_EDTA in a vortexer for 5 min, as described in reference 82. Samples were centrifuged at 5,000 rpm for 5 min, and 1 ml was transferred to a high-performance liquid chromatography (HPLC) vial and submitted for high-resolution LC-mass spectrometry (LC-MS) analysis. LC was performed with an Agilent 1260 Infinity HPLC system using an Acclaim Trinity Q1 column (2.1 mm by 100 mm, 3-μm particle size). The injection volume and the flow rate were 10 μl and 0.25 ml/min, respectively, during an isocratic elution using a mobile phase of 50 mM ammonium formate (pH 2.9; formic acid) for 5 min. Eluting species were detected by an Agilent 6530 Accurate-Mass quadrupole time of flight (Q-TOF) mass spectrometer equipped with a Jet Stream electrospray ion source in negative mode. The ion source settings were capillary voltage, 3,000 V; nozzle voltage, 2,000 V; fragmentor voltage, 180 V; drying gas and sheath gas temperature, 350°C; drying gas flow, 10 liters/min; sheath gas flow, 11 liters/min; and nebulizer pressure, 45 lb/in^2^. Glyphosate (C_3_H_8_NO_5_P) was observed in the samples with this LC-MS method as [M–H]^–^ at 168.0067 Da, with a retention time of 2.2 minutes. Glyphosate quantification was performed by preparing analytical curves using the area under the glyphosate extracted ion chromatogram peak of different standard solutions prepared from a 1.0-mg/ml glyphosate stock solution in water as follows: 1.25, 2.5, 5.0, 10, and 25 μg/ml glyphosate for samples collected from hives treated with 0.001% Roundup in sucrose syrup or water; and 50, 100, 200, 300, 400, and 500 μg/ml glyphosate for samples collected from hives treated with 0.1% Roundup in sucrose syrup or water. Quantification limits (QLs) were obtained by calculating the ratio between the standard deviation of the lower concentration used in the analytical curve and the slope of the analytical curve and then multiplying by 10. One sample from the 0.001% Roundup group collected at week 0, site 2 was excluded from the analyses due to contamination. The linear equations obtained from the analytical curves were used to calculate the concentration of glyphosate in the samples. The exact mass weighed for each sample was converted to volume, considering the density of sucrose syrup 1:1 (wt/vol) equal to 1.22 g/ml. Then, the concentrations obtained from the linear equation were corrected for the dilution factor.

### Statistical analyses.

For some oral and topical exposure experiments, comparisons of changes in bacterial abundance between control and treatment groups were performed using the nonparametric Kruskal-Wallis test followed by Dunn’s multiple-comparison test, if significant, in R version 3.5.2 (83). Principal coordinate analyses based on Bray-Curtis or weighted Unifrac dissimilarities were plotted using the R package “phyloseq” (84), and statistical tests were performed using pairwise permutational multivariate analysis of variance (PERMANOVA) tests with 999 permutations in QIIME 2 version 2019.10 (78). Comparisons of changes in recovered or surviving bees between control and treatment groups were performed using the chi-squared test followed by Bonferroni’s correction.

For some oral exposure experiments and all the field experiments, generalized linear mixed-effects models assuming Poisson regression were used to compare changes in bacterial abundances between control and treatment bees or hives, respectively, per sampling time. Treatment and sampling time were considered fixed effects and bees or bees nested within hives as random effects. Mixed models were fitted using the R package “lme4” (85), followed by *post hoc* tests using the R package “emmeans” (86).

For some topical exposure experiments and all the *Serratia* challenge experiments, comparisons of survival rates between control and treatment groups were performed using Kaplan-Meier survival curves and the Cox proportional hazards model implemented in the R package “survival” (52).

For some topical exposure experiments, dose-response models were fitted using the drm and LL.4 functions to fit and define the structure of the regression model, and the modelFit function was used to obtain a lack-of-fit test, which were all performed in the R package “drc” (87).

### Data availability.

All sequence data are available at NCBI BioProject PRJNA630698. Final tables and R scripts are available in GitHub (https://github.com/erickmotta/aem-2020). The other data generated during this study are included in this published article and its supplementary information files.

## Supplementary Material

Supplemental file 1
