# Supplementary material for: Oral or Topical Exposure to Glyphosate in Herbicide Formulation Impacts the Gut Microbiota and Survival Rates of Honey Bees
Source: Appl Environ Microbiol. 2020 Sep 1;86(18):e01150-20. doi: 10.1128/AEM.01150-20 (PMC7480383; doi:10.1128/AEM.01150-20)
Supplement: Supplemental file 1 [file AEM.01150-20-s0001.pdf]

Supplemental Material

Oral and topical exposure to glyphosate in herbicide formulation impact the gut microbiota and survival rates of honey bees

Erick V. S. Motta[1]*, Myra Mak[1], Tyler K. De Jong[1], J. Elijah Powell[1], Angela O'Donnell[1], Kristin J. Suhr[2], Ian M. Riddington[2], Nancy A. Moran[1]*

Affiliations:
[1]Department of Integrative Biology, University of Texas at Austin, Texas, USA
[2]Mass Spectrometry Facility, Department of Chemistry, University of Texas at Austin, Texas, USA

*Corresponding authors:
Erick V. S. Motta and Nancy A. Moran
2506 Speedway, Austin, TX 78712
Emails: erickvsm@utexas.edu and nancy.moran@austin.utexas.edu

This PDF file includes:

Figures S1 to S12
Tables S1 to S6
References

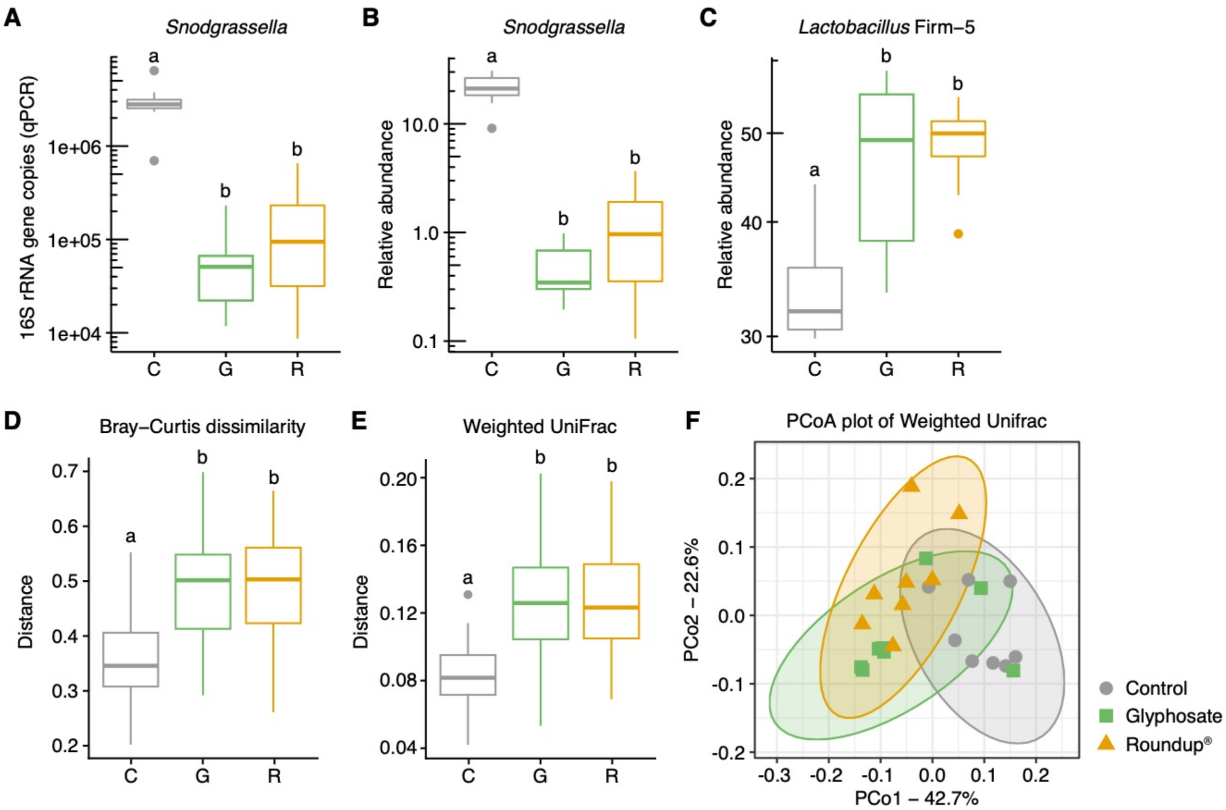

Figure S1. The effects of glyphosate and Roundup® formulation on the honey bee gut microbiota.
(A) *Snodgrassella* absolute abundance estimated by qPCR, and **(B)** *Snodgrassella* and **(C)** *Lactobacillus* Firm-5 relative abundances estimated by 16S rRNA amplicon sequencing in the guts of bees sampled from Control, Glyphosate and Roundup® groups (n = 8 for each group). **(D)** Bray-Curtis and **(E)** weighted UniFrac dissimilarity distances in gut communities among control bees versus between control bees and treatment bees. Groups with distinct letters are statistically different ($P < 0.05$, Kruskal-Wallis test followed by Dunn's multiple comparisons test). **(F)** Principal coordinate analysis of weighted UniFrac dissimilarity of gut community compositions of Control, Glyphosate and Roundup® groups (for significance, see Table S1).

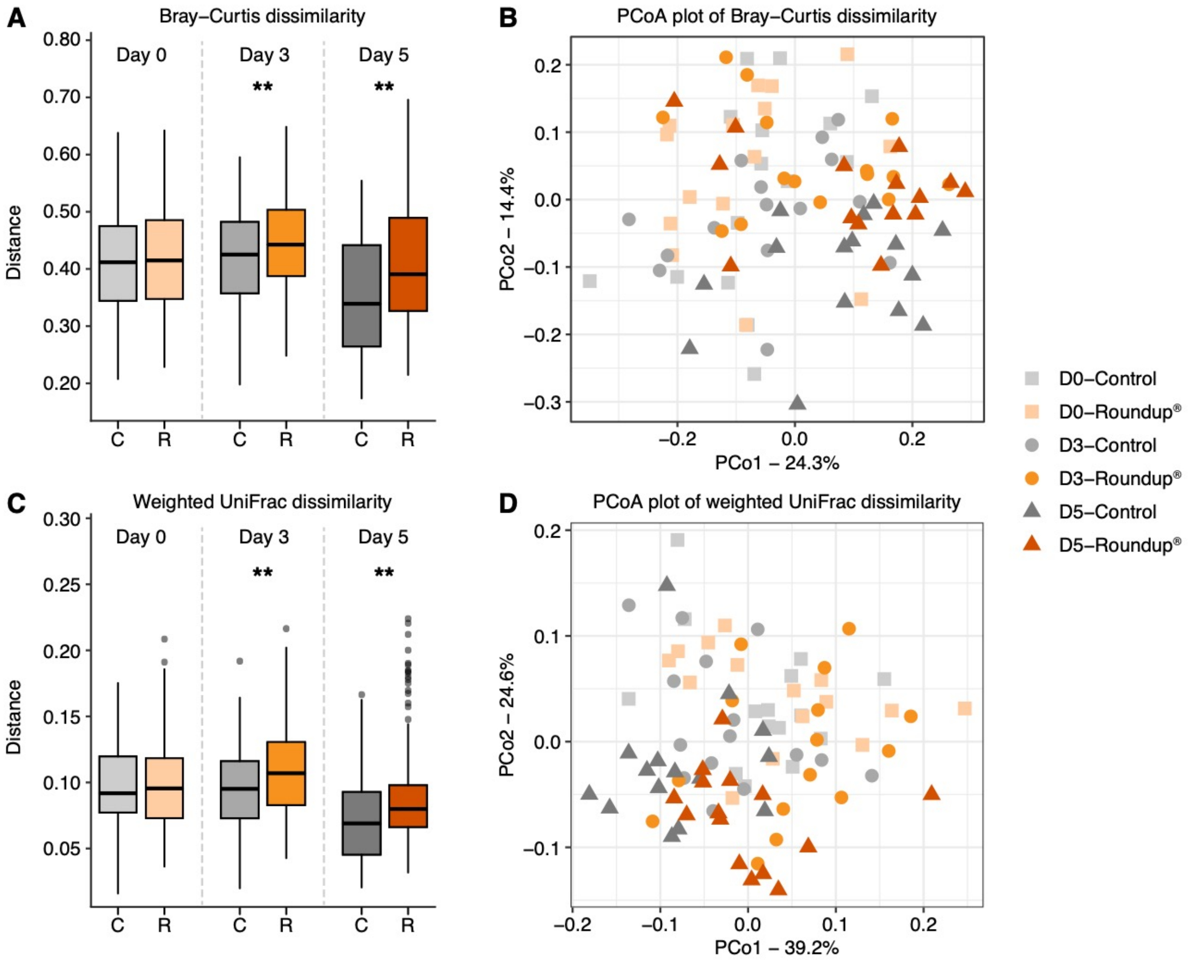

Figure S2. Changes in microbial diversity in bees orally exposed to 0.1% Roundup® formulation.

Boxplots show **(A)** Bray-Curtis and **(B)** weighted UniFrac dissimilarity distances in gut communities among control bees versus between control bees and treatment bees. $^{**}P < 0.01$, Kruskal-Wallis test followed by Dunn's multiple comparisons test. Principal coordinate analysis of **(C)** Bray-Curtis and **(D)** weighted UniFrac dissimilarities of gut community compositions of control and treatment groups, respectively. Pairwise Permanova test with 999 permutations was used to compare control and treatment samples per sampling time (for significance, see Table S2).

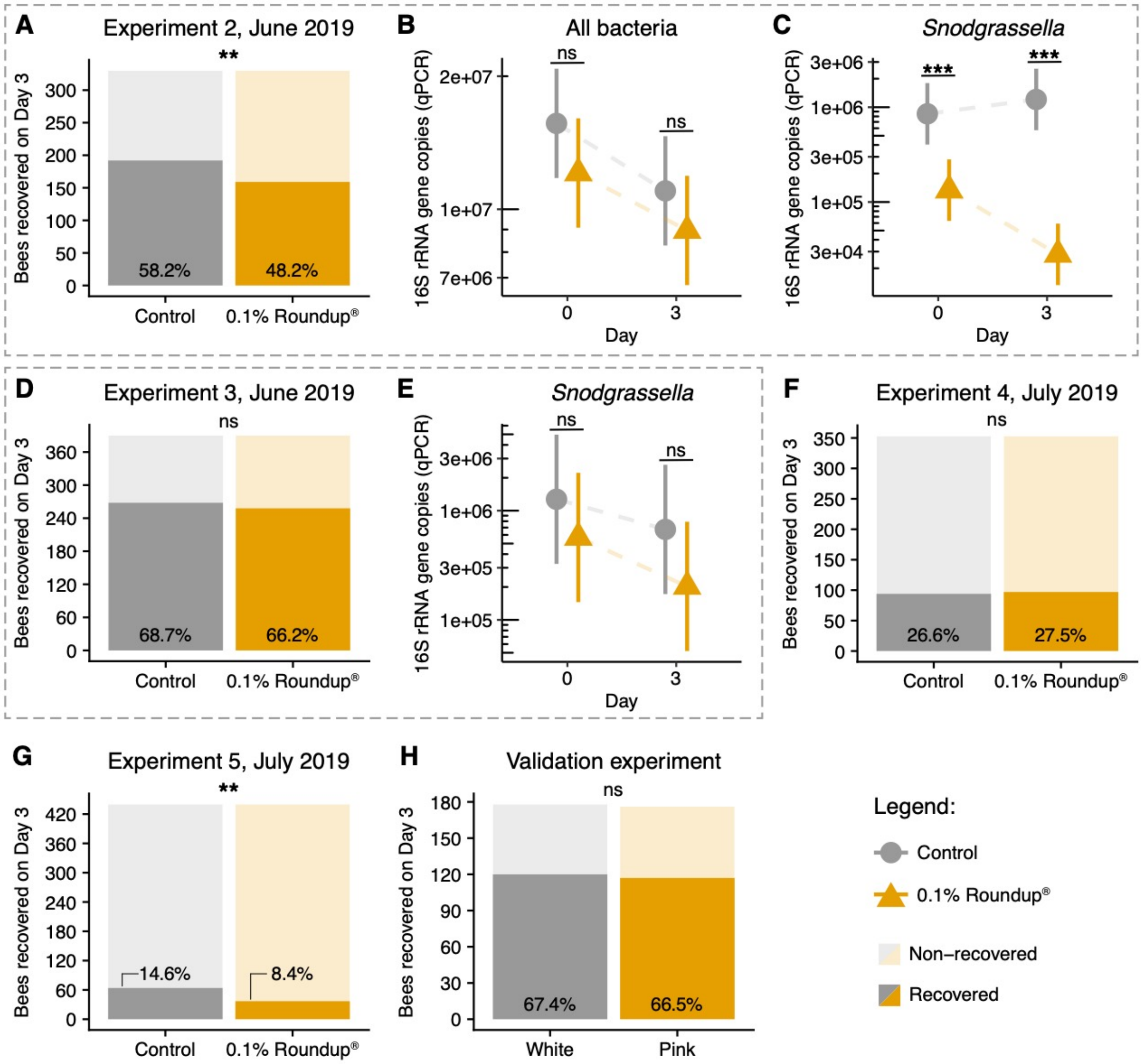

Figure S3. Recovery rates of honey bees orally exposed to 0.1% Roundup® formulation.

(A, B, C) Experiment 2; **(D, E)** Experiment 3; **(F, G)** Experiments 4 and 5; **(H)** Experiment to test color-bias in recovery experiments between the two colors used, pink and white. Stacked column graphs show the number of worker bees recovered from different hives at different seasons at day 3 post-treatment (** = $P < 0.01$, ns = nonsignificant difference, Chi-squared test). Scatter plots show total bacterial and *Snodgrassella* abundances in the guts of worker bees sampled at days 0 and 3 post-treatment of experiments 2 and 3 (n = 15 for each group and time point). For more details, see Table S3.

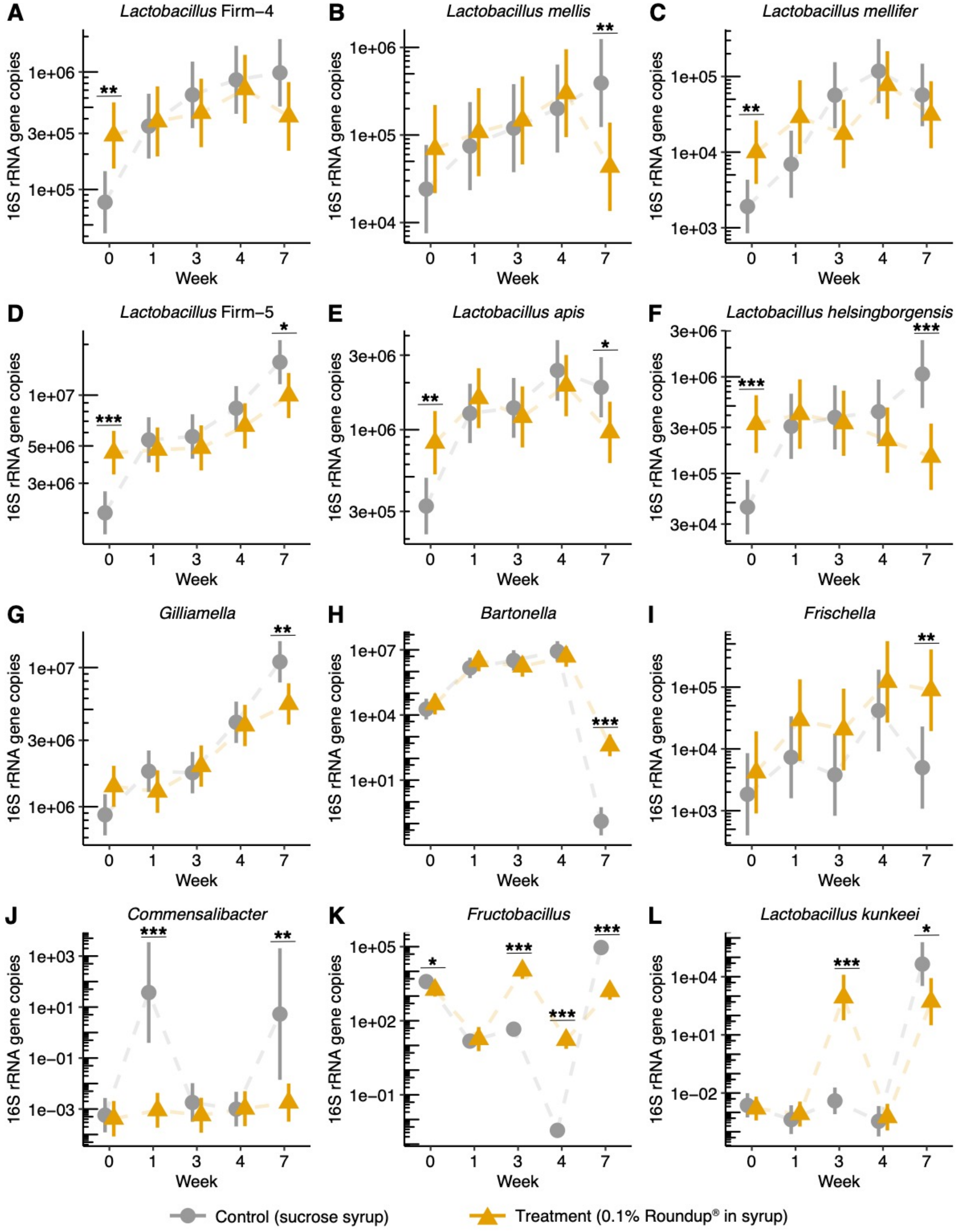

Figure S4. Gut microbial perturbations in honey bees from hives exposed to 0.1% Roundup® in sucrose syrup in site 1, 2018.

Scatter plots of estimates of absolute abundance for **(A)** *Lactobacillus* Firm-4, including **(B)** *Lactobacillus mellis* and **(C)** *Lactobacillus mellifer*; **(D)** *Lactobacillus* Firm-5, including **(E)** *Lactobacillus apis* and **(F)** *Lactobacillus helsingborgensis*; **(G)** *Gilliamella*; **(H)** *Bartonella*; **(I)** *Frischella*; **(J)** *Commensalibacter*; **(K)** *Fructobacillus*; and **(L)** *Lactobacillus kunkeei* in the guts of bees sampled from control (sucrose syrup) and treatment (0.1% Roundup® in syrup) groups on weeks 0, 1, 3, 4 and 7, with error bars of 95% confidence interval. Generalized linear mixed-effects models assuming Poisson regression were used to compare changes in bacterial abundances between control and treatment groups (n = 5 hives per group, 15 bees per hive) per sampling time. Mixed models were fitted using the package lme4 (1) and followed by *post hoc* tests using the package emmeans (2). *$P < 0.05$, **$P < 0.01$ and ***$P < 0.001$.

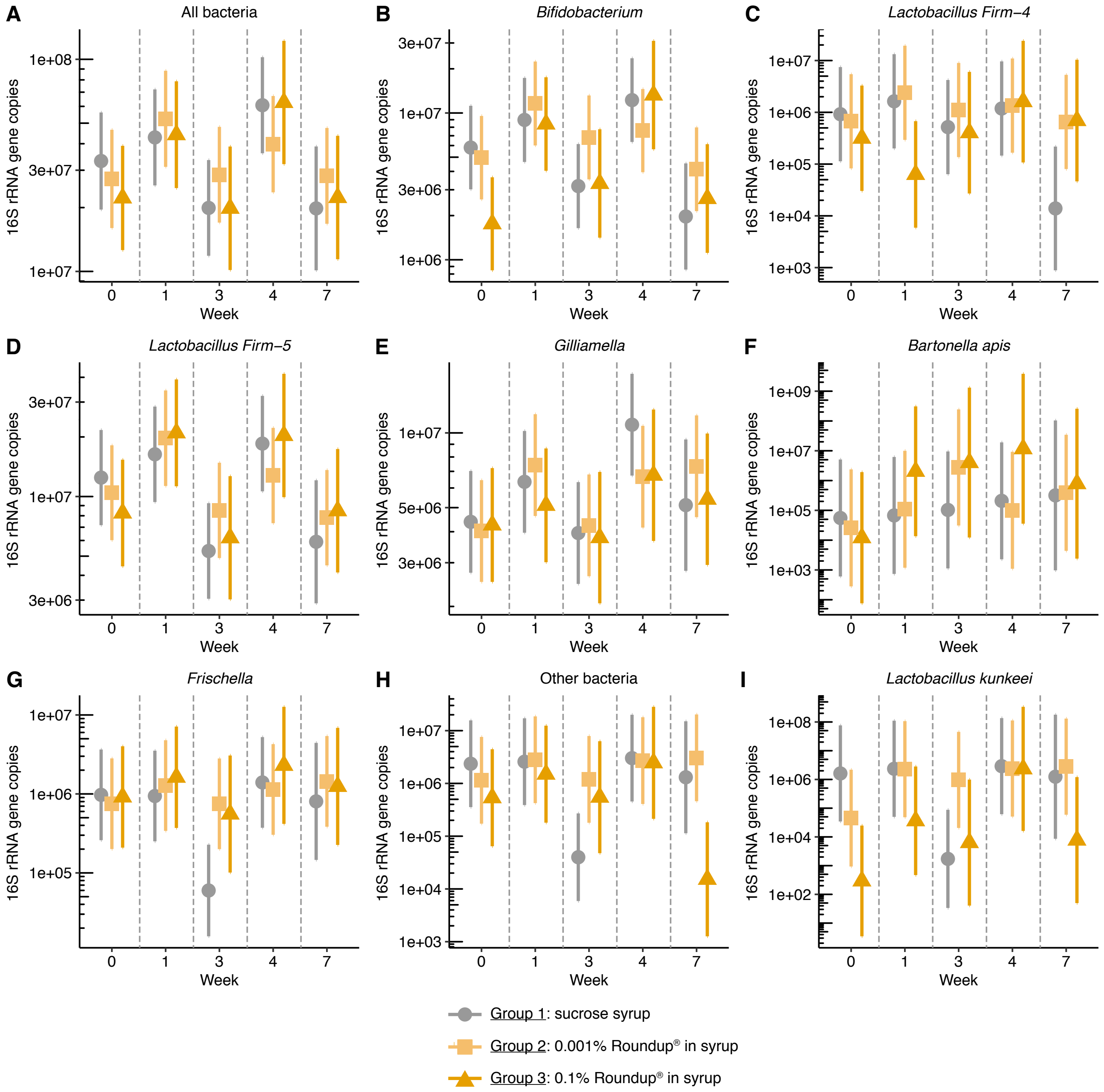

Figure S5. Gut microbial perturbations in honey bees from hives exposed to 0.001% or 0.1% Roundup® **in syrup in site 1, 2019.**

Scatter plots of estimates of absolute abundance for **(A)** total bacteria; **(B)** *Bifidobacterium*; **(C)** *Lactobacillus* Firm-4; **(D)** *Lactobacillus* Firm-5; **(E)** *Gilliamella*; **(F)** *Bartonella*; **(G)** *Frischella*; **(H)** and other bacteria, including **(I)** *Lactobacillus kunkeei* in the guts of bees sampled from control (sucrose syrup) and treatment (0.001% or 0.1% Roundup® in syrup) groups on weeks 0, 1, 3, 4 and 7, with error bars of 95% confidence interval. For group 1: n = 5 for weeks 0, 1, 3 and 4; n = 3 for week 7. For group 2: n = 5. For group 3: n = 4 for weeks 0 and 1; n = 3 for weeks 3, 4 and 7. Each hive is represented by 15 pooled bee guts.

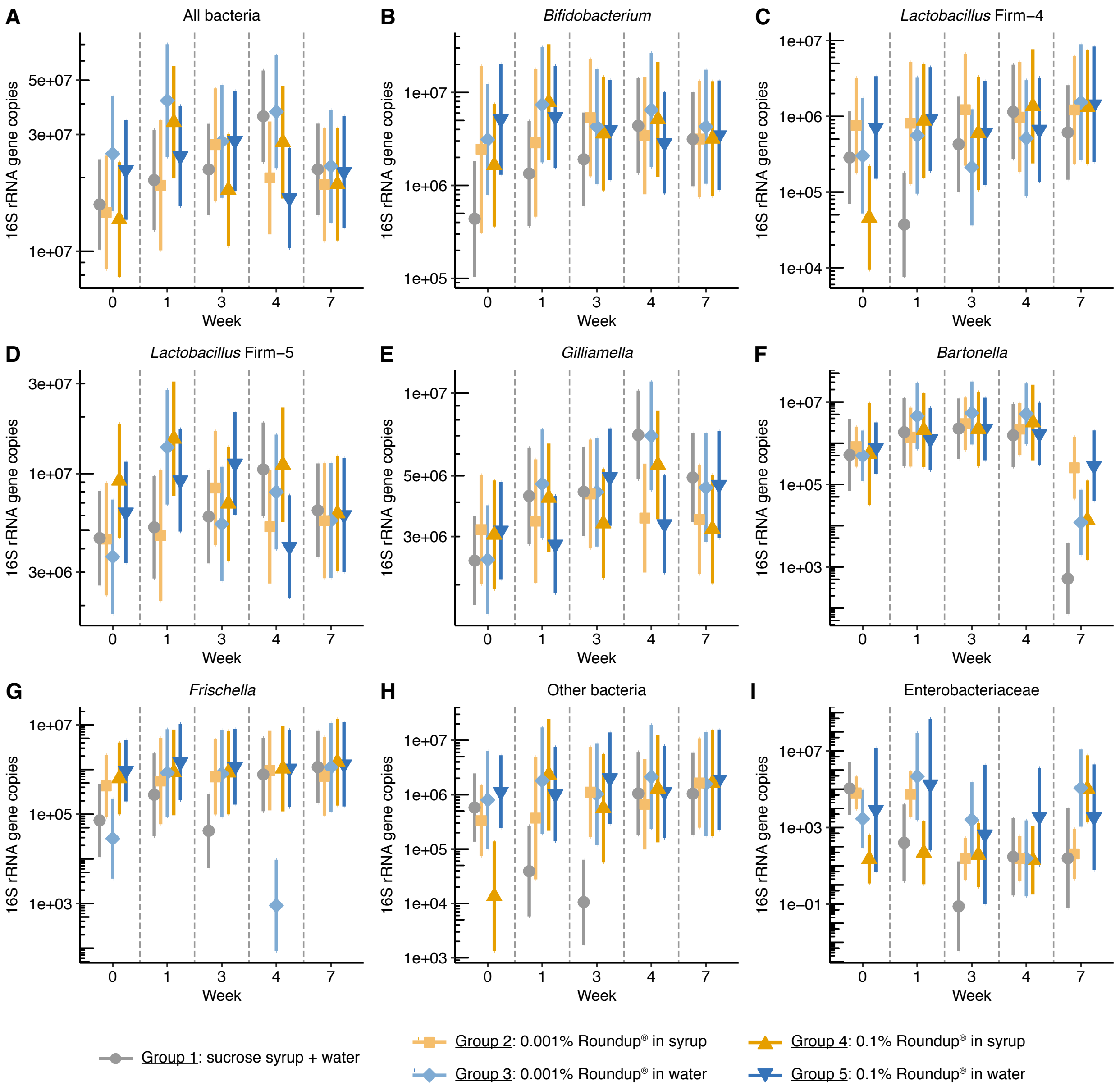

Figure S6. Gut microbial perturbations in honey bees from hives exposed to 0.001% or 0.1% Roundup® in syrup or water in site 2, 2019.
Scatter plots of estimates of absolute abundance for **(A)** total bacteria; **(B)** *Bifidobacterium*; **(C)** *Lactobacillus* Firm-4; **(D)** *Lactobacillus* Firm-5; **(E)** *Gilliamella*; **(F)** *Bartonella*; **(G)** *Frischella*; **(H)** and other bacteria, including **(I)** Enterobacteriaceae in the guts of bees sampled from control (sucrose syrup + water) and treatment (0.001% or 0.1% Roundup® in syrup or water) groups on weeks 0, 1, 3, 4 and 7, with error bars of 95% confidence interval. For group 1: n = 6 for weeks 0, 3, 4 and 7; n = 5 for week 1. For group 2: n = 4 for weeks 0, 3, 4 and 7; n = 3 for week 1. For groups 3 and 4: n = 4. For group 5: n = 5 for weeks 0, 1, 3 and 4; n = 4 for week 7 Each hive is represented by 15 pooled bee guts.

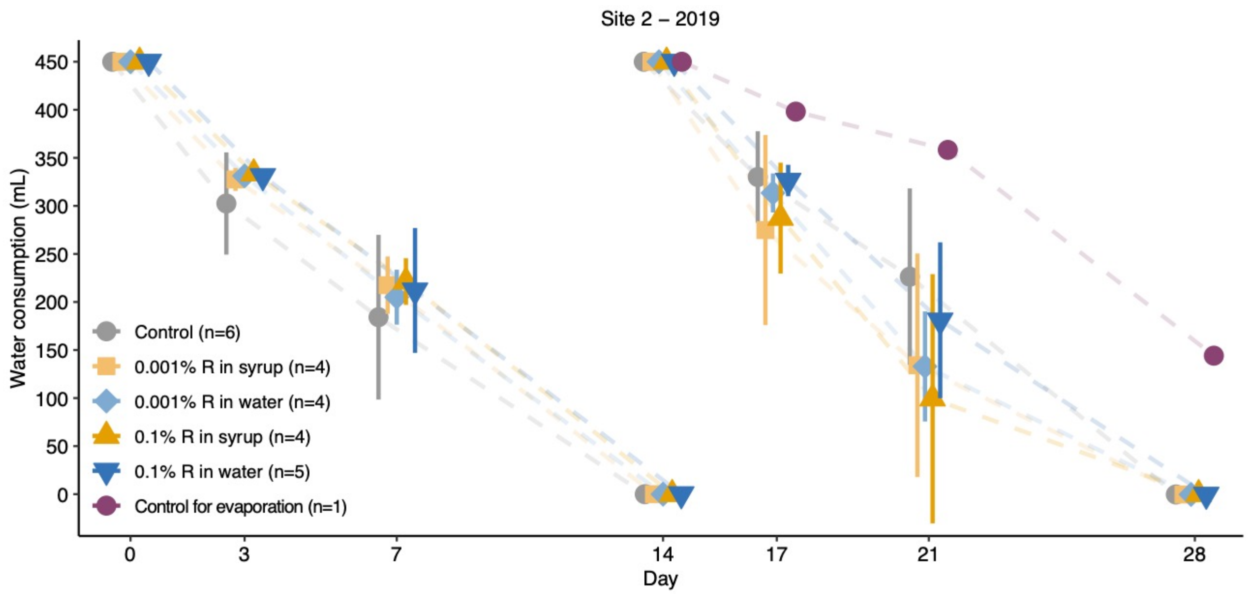

Figure S7. Estimates of water consumption in hives from site 2, 2019.
Scatter plots of water or formulation in water consumption in control and treatment hives, respectively, for a period of four weeks. Water or formulation in water were provided to each hive in a glass bottle with punched cap connected to a plastic boardman and attached to the hive entry. Water evaporation was evaluated after Week 2 by adding the same apparatus in an empty hive.

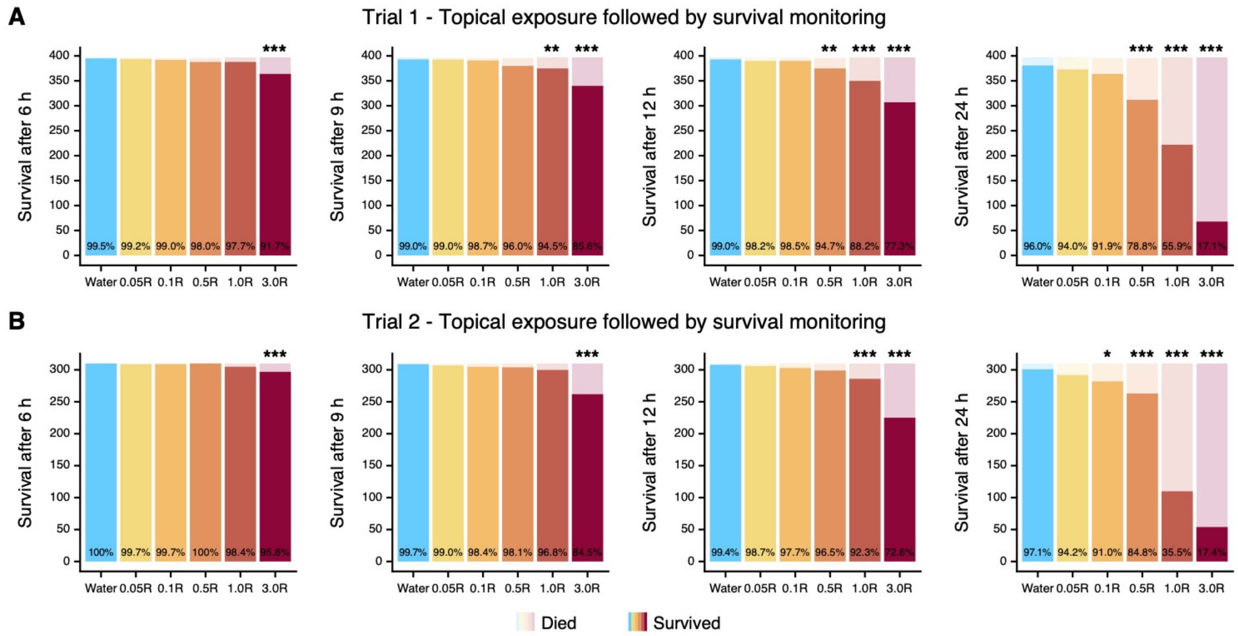

Figure S8. Survival rates of honey bees topically exposed to Roundup® formulation under laboratory conditions.

(A) Trial 1, also shown in Figure 6. **(B)** Trial 2. In both trials, survival rates were measured 6, 9, 12 and 24 hours after spraying worker bees with different concentrations of a glyphosate-based formulation in water (n = 8-9 cup cages per group, 35-40 bees per cup cage). $^{*}P < 0.05$, $^{**}P < 0.01$, $^{***}P < 0.001$, Chi-squared test followed by Bonferroni correction

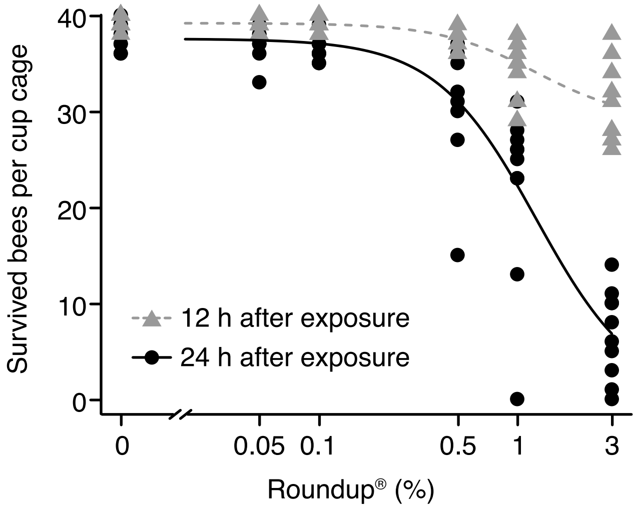

Figure S9. Dose-response curves for survival rates of honey bees topically exposed to different concentrations of Roundup® formulation.

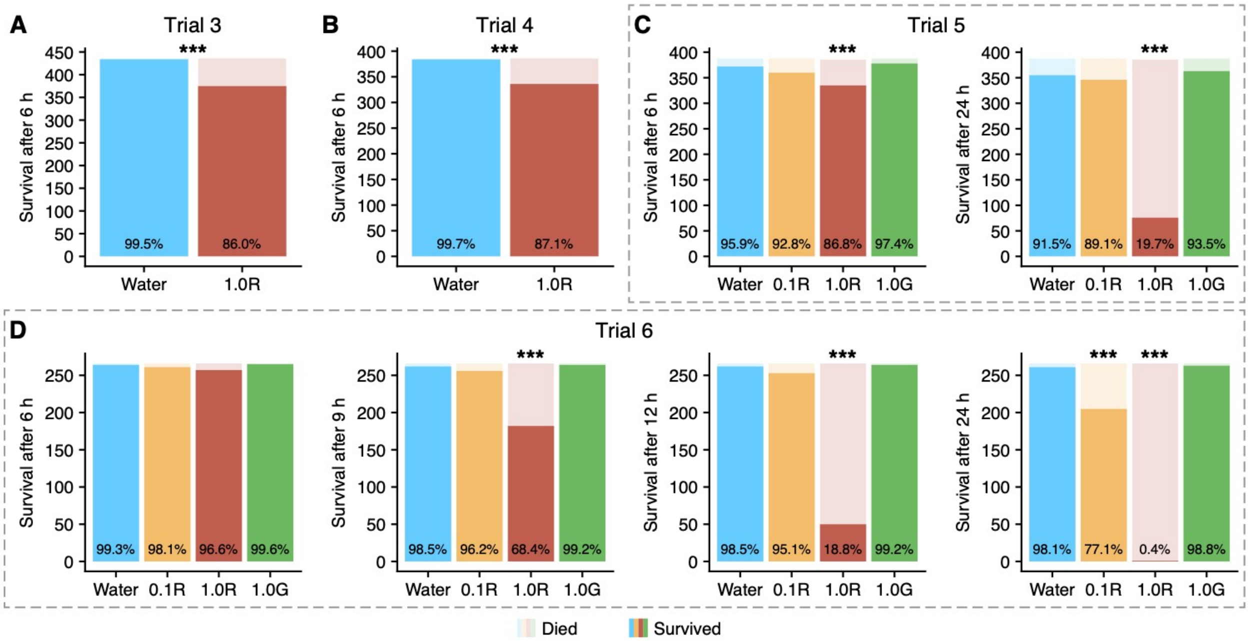

Figure S10. Survival rates of honey bees topically exposed to glyphosate and Roundup® formulation under laboratory conditions.

In **(A)** and **(B)**, worker bees were split into 2 groups which were sprayed with either tap water or 1.0% Roundup® formulation in water. Survivorship was monitored for 6 hours under laboratory conditions (n = 10 cup cages per group, 35-40 bees per cup cage). In **(C)** and **(D)**, worker bees were split into 4 groups which were sprayed with either tap water, 0.1% Roundup® in water, 1.0% Roundup® in water or 1.0% glyphosate in water. Survivorship was monitored for 24 hours under laboratory conditions (n = 10 cup cages per group, 35-40 bees per cup cage). ***$P < 0.001$, Chi-squared test followed by Bonferroni correction.

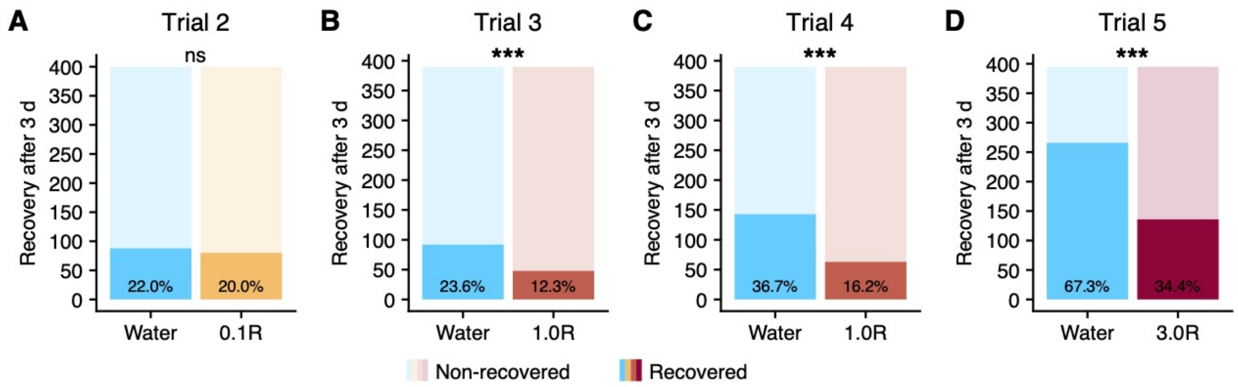

Figure S11. Recovery rates of honey bees topically exposed to a glyphosate-based formulation under hive conditions.

Worker bees were split into two groups which were marked on the thorax with blue or green paint. In each trial, groups were sprayed with either water or a specific concentration of a glyphosate-based formulation in water, **(A)** 0.1%, **(B)** and **(C)** 1.0%, **(D)** 3.0%, and released back to their hive (n = 10 cup cages per group, 35-40 bees per replicate). All marked bees were recovered on day 3 post-spray. ns, non-significant, ***$P < 0.001$, Chi-squared test followed by Bonferroni correction.

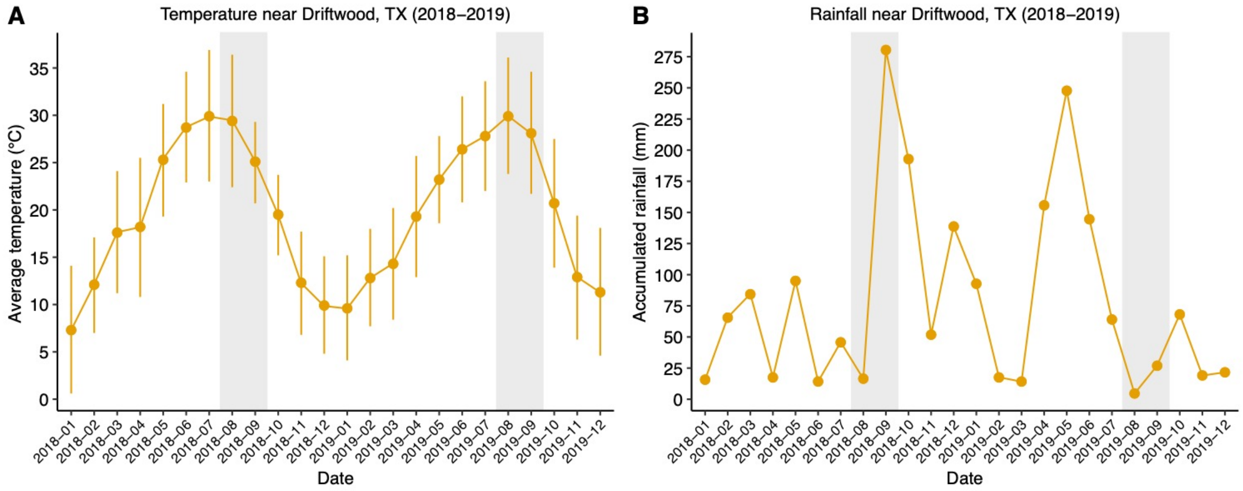

Figure S12. Climate data for the region of Driftwood, TX, in the period of 2018-2019.
(A) Monthly averages of temperature and **(B)** monthly accumulated rainfall data obtained from the automatic station of climate data collection Dripping Springs 6 E, TX US (USC00412585). Source: National Centers for Environmental Information (NCEI, www.ncdc.noaa.gov).

Table S1. Permanova results of Bray-Curtis and Weighted UniFrac dissimilarities for gut community compositions of age-controlled honey bees orally exposed to treatments under laboratory conditions.

**Bray-Curtis Dissimilarity**						
method name	PERMANOVA					
test statistic name	pseudo-F					
sample size	24					
number of groups	3					
test statistic	3.70252					
p-value	0.001					
number of permutations	999					
Pairwise permanova results						
Group 1	Group 2	Sample size	Permutations	pseudo-F	p-value	q-value
Control	Glyphosate	16	999	3.90132027	0.001	0.0015
Control	Roundup	16	999	6.14771957	0.001	0.0015
Glyphosate	Roundup	16	999	1.68063484	0.081	0.081

**Weighted UniFrac Dissimilarity**						
method name	PERMANOVA					
test statistic name	pseudo-F					
sample size	24					
number of groups	3					
test statistic	4.46616					
p-value	0.001					
number of permutations	999					
Pairwise permanova results						
Group 1	Group 2	Sample size	Permutations	pseudo-F	p-value	q-value
Control	Glyphosate	16	999	4.42337273	0.008	0.012
Control	Roundup	16	999	7.99143437	0.001	0.003
Glyphosate	Roundup	16	999	2.21440432	0.053	0.053

Table S2. Permanova results of Bray-Curtis and weighted UniFrac dissimilarities for gut community compositions of honey bees orally exposed to 0.1% Roundup® and recovered at days 0, 3 and 5 post-treatment.

**Bray-Curtis Dissimilarity**						
method name	PERMANOVA					
test statistic name	pseudo-F					
sample size	90					
number of groups	6					
test statistic	2.89958					
p-value	0.001					
number of permutations	999					
Pairwise permanova results						
Group 1	Group 2	Sample size	Permutations	pseudo-F	p-value	q-value
D0-C	D0-T	30	999	0.618492438	0.815	0.815
D0-C	D3-C	30	999	0.638190516	0.76	0.8143
D0-C	D3-T	30	999	1.76256901	0.08	0.1
D0-C	D5-C	30	999	4.137791281	0.001	0.003
D0-C	D5-T	30	999	3.955942596	0.001	0.003
D0-T	D3-C	30	999	1.494438736	0.136	0.1569
D0-T	D3-T	30	999	2.002209517	0.035	0.0525
D0-T	D5-C	30	999	5.635417966	0.001	0.003
D0-T	D5-T	30	999	4.685699197	0.002	0.0043
D3-C	D3-T	30	999	2.573856554	0.003	0.0056
D3-C	D5-C	30	999	2.631917222	0.016	0.0267
D3-C	D5-T	30	999	4.150742984	0.001	0.003
D3-T	D5-C	30	999	4.982446668	0.001	0.003
D3-T	D5-T	30	999	1.791646927	0.08	0.1
D5-C	D5-T	30	999	3.752390141	0.002	0.0043

**Weighted UniFrac Dissimilarity**						
method name	PERMANOVA					
test statistic name	pseudo-F					
sample size	90					
number of groups	6					
test statistic	3.91951					
p-value	0.001					
number of permutations	999					
Pairwise permanova results						
Group 1	Group 2	Sample size	Permutations	pseudo-F	p-value	q-value
D0-C	D0-T	30	999	0.113830359	0.981	0.981
D0-C	D3-C	30	999	1.283408518	0.277	0.2968
D0-C	D3-T	30	999	1.58824016	0.186	0.2325
D0-C	D5-C	30	999	6.130568942	0.001	0.0025
D0-C	D5-T	30	999	6.403509713	0.001	0.0025
D0-T	D3-C	30	999	2.045786614	0.075	0.1023
D0-T	D3-T	30	999	1.34839217	0.227	0.2619
D0-T	D5-C	30	999	7.337864044	0.002	0.0043
D0-T	D5-T	30	999	7.04935978	0.001	0.0025
D3-C	D3-T	30	999	3.877954422	0.007	0.0131
D3-C	D5-C	30	999	2.252614238	0.07	0.1023
D3-C	D5-T	30	999	5.818113513	0.001	0.0025
D3-T	D5-C	30	999	7.831137128	0.001	0.0025
D3-T	D5-T	30	999	3.216409879	0.018	0.03
D5-C	D5-T	30	999	5.586797101	0.001	0.0025

Table S3. Oral exposure experiments followed by hive recovery.

Experiment	Season	Hive ID	Group treatment	Color	Released bees
1	Fall, 2018	8, Imperial*	Sucrose syrup	White	348
			0.1% Roundup® in syrup	Pink	348
2	Spring, 2019	9, Alsatian	Sucrose syrup	White	330
			0.1% Roundup® in syrup	Pink	330
3	Spring, 2019	8, Labrador	Sucrose syrup	Pink	390
			0.1% Roundup® in syrup	White	390
4	Summer, 2019	8, Labrador	Sucrose syrup	Pink	353
			0.1% Roundup® in syrup	White	353
5	Summer, 2019	8, Labrador	Sucrose syrup	White	440
			0.1% Roundup® in syrup	Pink	440
Validation	Summer, 2019	9, Alsatian	Sucrose syrup	Pink	178
			Sucrose syrup	White	176

* This hive died in Winter, 2018.

Table S4. Treatment scheme performed in the field experiments.

Time	Site	Group	Number of hives	Treatment	Week					
					0	1	2	3	4	7
Aug/Sep 2018	1	Control	5	Sucrose syrup	X	X	X	X		
		0.1R-S	5	0.1% Roundup® in syrup	X	X	X	X		
Aug/Sep 2019	1	Control	5	Sucrose syrup	X	X	X	X		
		0.001R-S	5	0.001% Roundup® in syrup	X	X	X	X		
		0.1R-S	4	0.1% Roundup® in syrup	X	X	X	X		
	2	Control	6	Sucrose syrup	X					
				Water	X		X			
		0.001R-S	4	0.001% Roundup® in syrup	X					
				Water	X		X			
		0.1R-S	4	0.1% Roundup® in syrup	X					
				Water	X		X			
		0.001R-W	4	Sucrose syrup	X					
				0.001% Roundup® in water	X		X			
		0.1R-W	5	Sucrose syrup	X					
				0.1% Roundup® in water	X		X			

Table S5. Topical exposure experiments performed under laboratory conditions.

Experiment	Season	Hive ID	Group treatment	# Cup cages	# Bees	Survival monitoring
1	Spring, 2019	3, Firefly	Water	12	436	6 h post-spray
			1.0% Roundup® in water	12	436	
2	Summer, 2019	3, Firefly	Water	10	385	6 h post-spray
			1.0% Roundup® in water	10	385	
3	Summer, 2019	3, Firefly	Water	10	388	6 and 24 h post-spray
			0.1% Roundup® in water	10	386	
			1.0% Roundup® in water	10	388	
			1.0% glyphosate in water	10	388	
4	Summer, 2019	0, Avocado	Water	7	266	6, 9, 12 and 24 h post-spray
			0.1% Roundup® in water	7	266	
			1.0% Roundup® in water	7	266	
			1.0% glyphosate in water	7	266	
5	Fall, 2019	1, Leviathan	Water	8	310	6, 9, 12 and 24 h post-spray
			0.05% Roundup® in water	8	310	
			0.1% Roundup® in water	8	310	
			0.5% Roundup® in water	8	310	
			1.0% Roundup® in water	8	310	
			3.0% Roundup® in water	8	310	
6	Fall, 2019	6, Pyrenees	Water	10	397	6, 9, 12 and 24 h post-spray
			0.05% Roundup® in water	10	397	
			0.1% Roundup® in water	10	397	
			0.5% Roundup® in water	10	397	
			1.0% Roundup® in water	10	397	
			3.0% Roundup® in water	10	397	

Table S6. Topical exposure experiments followed by hive recovery.

Experiment	Season	Hive ID	Group treatment	Color	Bees released
1	Fall, 2019	2, Newfoundland	Tap water	Green	400
			0.1% Roundup® in water	Blue	400
2	Fall, 2019	2, Newfoundland	Tap water	Green	390
			1.0% Roundup® in water	Blue	390
3	Fall, 2019	2, Newfoundland	Tap water	Green	390
			1.0% Roundup® in water	Blue	390
4	Fall, 2019	2, Newfoundland	Tap water	Green	395
			3.0% Roundup® in water	Blue	395
5	Fall, 2019	6, Pyrenees	Tap water	White	260
			0.05% Roundup® in water	Green	260
			0.1% Roundup® in water	Orange	260
			0.5% Roundup® in water	Pink	260
			1.0% Roundup® in water	Yellow	260
			3.0% Roundup® in water	Blue	260

References

1. Bates D, Mächler M, Bolker B, Walker S. 2015. Fitting linear mixed-effects models using lme4. J Stat Softw 67:1–48.

2. Lenth R, Singmann H, Love J, Buerkner P, Herve M. 2019. emmeans: Estimated Marginal Means, aka Least-Squares Means.
